# Integrating bulk RNA-seq, scRNA-seq, and spatial transcriptomics data to identify novel post-translational modification-related molecular subtypes and therapeutic responses in hepatocellular carcinoma

**DOI:** 10.1186/s12935-025-03964-y

**Published:** 2025-10-03

**Authors:** Shiling Chen, Yunjie Li, Jichang Hu, Heli Li, Chen Hu, Jinzhu Zhao, Hong Qian, Shuya Bai, Zhouping Tang, Yangyang Feng

**Affiliations:** 1 Department of Neurology, Tongji Hospital of Tongji Medical College, Huazhong University of Science and Technology, Wuhan, 430030 Hubei China; 2 Division of Child Healthcare, Department of Pediatrics, Tongji Hospital, Tongji Medical College, Huazhong University of Science and Technology, Wuhan, 430030 China; 3 Department of Pathophysiology, School of Basic Medicine, Key Laboratory of Education Ministry of China for Neurological Disorders, Tongji Medical College, Huazhong University of Science and Technology, Wuhan, China; 4 Department of Gastroenterology and Hepatology, Tongji Hospital, Tongji Medical College, Huazhong University of Science and Technology, Wuhan, 430030 China

**Keywords:** Hepatocellular carcinoma, Post-translational modification, Multi-omics analysis, Immunotherapy

## Abstract

**Background:**

Hepatocellular carcinoma (HCC) poses considerable difficulties regarding the prognosis and the assessment of treatment efficacy. Additionally, while it is recognized that post-translational modification (PTM) plays a crucial role in modulating HCC progression, their specific prognostic implications in HCC have not been thoroughly investigated.

**Methods:**

21 types of PTM (acetylation, succinylation, malonylation, crotonylation, β-hydroxybutyrylation, lactylation, palmitoylation, myristoylation, SUMOylation, NEDDylation, ISGylation, ATG8ylation, FAT10ylation, UFMylation, methylation, glycosylation, biotinylation, S-nitrosylation, phosphorylation, ubiquitination, deubiquitination) were generated consensus cluster. Then, WGCNA was utilized to identify module genes. Finally, a machine learning approach was employed to create PTM.score.

**Results:**

This analysis revealed two distinct subtypes of PTMs, each characterized by unique molecular signatures. By integrating different categories of genes, including prognosis-related DEGs, module genes, and PTM-related genes, 15 hub genes were identified, and a PTM.score was developed. PTM.score was rigorously validated across independent external cohorts (TCGA-LIHC, LIRI-JP, GSE10143, GSE14520, GSE27150, GSE36376, and GSE76427) and an in-house cohort, demonstrating its reliability and potential applicability. In addition, patients categorized with a low PTM.score displayed a TME that was more actively engaged, which corresponded with a poor prognosis. Furthermore, these patients demonstrated a high level of responsiveness to immunotherapy interventions. Furthermore, an examination using scRNA-seq and spatial transcriptomics indicated that patients with low PTM.score exhibited heightened cell proliferation and malignancy.

**Conclusion:**

This novel PTM-related prognostic signature could effectively assess the prognosis and therapeutic responses of HCC patients, providing new perspectives for individualized treatment for the patient population.

**Supplementary Information:**

The online version contains supplementary material available at 10.1186/s12935-025-03964-y.

## Introduction

Hepatocellular carcinoma (HCC) presents with complex clinical symptoms and is associated with a poor prognosis, while its rates of occurrence and mortality continue to rise [1]. Treatment options for HCC include surgery, radiotherapy, chemotherapy, and targeted therapy, but the overall survival rate is low, especially for advanced patients [2]. Therefore, it is imperative explore more effective treatment strategies. With the development of immunology, immunotherapy has gradually become a research hotspot in the treatment of HCC, providing new choices for HCC patients to maintain long-term remission [3]. In light of the potential that ICIs hold for cancer treatment, it is crucial to acknowledge that the current response rate to these therapies is still under 30% [4–6]. This limited efficacy serves as a significant obstacle to the broader implementation of ICIs in clinical settings. The relatively low percentage of patients who benefit from these treatments raises questions about their overall effectiveness and highlights the need for further research. Enhancing response rates could be essential for encouraging greater acceptance and integration of ICIs into standard treatment protocols for cancer. Consequently, there is a pressing need for the identification of novel molecular markers, which would facilitate the development of new prognostic models and provide improved reference methods for the prognostic and therapeutic responses monitoring of HCC.

Post-translational modifications (PTMs) are crucial in modulating various aspects of protein biology, including their functional capabilities, levels of expression, and interactions with other molecular entities [7]. By altering the intrinsic characteristics and stability of proteins, these modifications help determine how proteins behave within a cellular context. This regulatory capacity underlines the importance of PTMs in the overall dynamics of cellular processes and highlights their role in influencing protein functionality across different biological systems [8–10]. These modifications have been identified as essential regulatory mechanisms in the field of cancer biology, where they particularly influence processes such as metabolic reprogramming and immune responses [11, 12]. Ubiquitination is critical PTM that oversees protein degradation and regulates cellular processes. This occurs through pathways like the ubiquitin-proteasome system (UPS) or lysosomal degradation, highlighting the importance of ubiquitination in maintaining cellular homeostasis and influencing cancer development [13]. TRIB2 expression is upregulated in liver cancer cells, where it acts with E3 ubiquitin ligases to regulate the protein stability of downstream effectors [14]. SIRT6 upregulates FOXO3 while increasing FOXO3 ubiquitination, thereby reducing its stability [15]. Clinically, CCT3 is positively correlated with YAP and TFCP2, and elevated levels of the CCT3-YAP-TFCP2 axis may be key to regulating the prognosis of malignant HCC [16]. This relationship indicates that the interplay between PTM can significantly influence HCC development. Moreover, the findings highlight this regulatory axis as a potentially valuable therapeutic target in HCC. By targeting this mechanism, new strategies can be developed that may enhance treatment efficacy and improve patient outcomes in HCC management.

Despite this, gaining a thorough insight into the relationship between PTM and HCC continues to be challenging, and a more in-depth exploration of how PTMs influence changes in the immune microenvironment is necessary. To address these knowledge deficiencies, we incorporated 21 different types of PTMs to seek out potential biomarkers for immunotherapy related to HCC. Initially, an HCC subtype was developed using the genes related to PTM. Following this, hub genes were pinpointed by employing WGCNA, PTM genes and analyzing DEGs and prognosis among the subtypes. By utilizing advanced machine learning algorithms, we have developed an innovative metric known as PTM.score, which serves the purpose of predicting therapeutic efficacy and prognosis specifically in HCC patients. Our research findings indicate that PTM.score excels in its ability to forecast clinical outcomes in individuals diagnosed with HCC. This significant capability underscores the importance of establishing a reliable framework that can enhance risk stratification and support informed decision-making regarding immunotherapeutic strategies in diverse HCC patient populations.

## Materials and methods

### Data gathering

Firstly, the transcriptome data from TCGA-LIHC, ICGC (LIRI-JP), GSE10143 [17], GSE14520 [18], GSE27150, GSE36376 [19], and GSE76427 [20] datasets were downloaded. In cases where multiple probes corresponded to a single gene, the expression level for that gene was determined by averaging the expression values of the various probes. To address batch effects between different databases, the FPKM format data from the TCGA-LIHC dataset was converted to TPM format data and merged with the TCGA-LIHC dataset and the ICGC (LIRI-JP), GSE10143, GSE14520, GSE27150, GSE36376, and GSE76427 datasets using the ComBat algorithm in the R package “sva“ [21]. Furthermore, we also assembled a collection of 7 immunotherapy cohorts, which include 5 focused on melanoma immunotherapy (Van Allen [22], Nathanson [23], GSE35640 [24], GSE78220 [25], GSE91061 [26]) and a metastatic urothelial cancer cohort (IMvigor210 cohort [27]). In addition to these, we incorporated an HCC immunotherapy cohort (GSE140901 [28]). Together, these datasets represent a diverse range of conditions and treatments, facilitating a thorough exploration of immunotherapy responses. Furthermore, PTM related genes were systematically curated through comprehensive literature examination (Supplementary Table 1).

## Consensus clustering

Cluster analysis is an analysis method that groups similar samples into a group through static classification, such that samples in different groups have differences, and samples in the same group have the same attributes. We used “ConsensusClusterPlus” to identify subtypes by PTM expression profiles [29]. PCA was used to evaluate the distribution differences patients in different subtypes. Kaplan-Meier survival curves compared the overall survival (OS) between different subtype patients and the statistical significance was assessed by log rank tests.

## DEGs screening and enrichment analysis

Consequently, DEGs were identified based on a significance threshold set at FDR < 0.01, ensuring that the findings were statistically significant and minimizing the likelihood of false positives. To further understand the possible biological functions and mechanisms of the key genes in HCC, we used the “clusterProfiler” package for GO and KEGG of the screened key genes [30]. During the analysis process, we retained the enriched items according to the significance level (*P* < 0.05) to ensure the reliability and accuracy of the results.

## Screening key genes based on WGCNA

We use the “WGCNA” package to screen out the key genes related to different subtypes in HCC. First, we selected the genes with 25% high variance, and the correlation matrix was constructed and the weighted adjacency matrix was generated by quantifying the correlation between each gene the cell phenotype through the gene expression spectrum. Second, the power exponent and the scale-free topological fitting index ^2^ = 0.85 were automatically calculated the function pickSoftThreshold and the function softConnectivity, respectively, and the soft threshold parameter was set so that the subsequent network construction was more consistent with the scale-free characteristics. Further, the topological overlap matrix was generated, the correlation degree between genes was calculated, and the hierarchical clustering tree of genes was obtained according to this. Finally, the clustering was divided, and different gene co-expression modules were obtained, the function verboseScatterplot was used to the correlation between gene expression and trait relatedness and gene expression and module relatedness, and the genes with GS value (> 0.80) and high MM value (> 0.85) were defined as the threshold for identifying key genes in the. Finally, the module most related to each subtype and the most important key genes in the module were obtained [31].

## Identification of Ptm.score

The TCGA-HCC dataset served as the foundational dataset for the construction of the risk model, while the datasets ICGC (LIRI-JP), GSE1014317, GSE1452018, GSE27150, GSE3637619, and GSE76427 were employed to validate the findings within the cohort. The methodology for generating the signature proceeded through several key steps: First, DEGs, prognostic, and WGCNA were conducted among the various subtypes. This initial analysis aimed to discern the significant genetic variations that may influence prognosis and therapeutic approaches. Next, a comprehensive integration of ten distinct machine learning algorithms was executed. This step was crucial for exploring different methodologies and determining which algorithms might provide the best predictive performance in the context of the model being developed. Finally, the C-index for dataset was calculated.

### Evaluation and analysis of TME

7 algorithms were used to analyze the TME for HCC. The distribution of cell types was depicted using the “pheatmap” R package in molecular subtype and high PTM.score and low PTM.score groups [32]. In addition, we the differences in the expression levels of immunomolecules in high PTM.score and low PTM.score groups.

## Gene sets enrichment analysis (GSEA)

All genes were sequenced according to the fold change of differential gene genes between subtypes, which was used to calculate enrichment scores in the following steps. A distribution of multiple random enrichment scores was generated by permutation of the gene set, which is used to estimate the statistical significance of the enrichment scores.

## scRNA-seq and spatial transcriptomics

The description of scRNA-seq data (GSE242889 [33]) and spatial transcriptomics (HRA000437 [34]) in the Supplementary material.

### RT-qPCR (Real-time quantitative PCR)

This study was approved by the Ethics Committee of Tongji Hospital of Tongji Medical College, Huazhong University of Science and Technology. All HCC samples were collected on condition of informed consent (TJ-2024-105). HCC tissue samples (*n* = 50) were obtained from patients at the Tongji Hospital. Total RNA was extracted from HCC tissue using TRIzol reagent (Invitrogen). Complementary DNA synthesis and subsequent qRT-PCR were integrated into a single step workflow.

### Immunohistochemistry (IHC)

FFPE tissue Sect. (5 μm) from HCC were deparaffinized, rehydrated, and subjected to heat-induced antigen retrieval in citric acid buffer (pH 6.0). Endogenous peroxidase activity was blocked, followed by incubation with primary antibodies against. After incubation with a biotinylated secondary antibody and streptavidin-HRP, signal was developed using DAB substrate, followed by counterstaining with hematoxylin.

### Statistics

R version 4.2.0 was the tool used for all bioinformatics analyses. We used the Kruskal-Wallis test for non-parametric data and the Wilcoxon test for comparisons between two independent samples and multiple samples, respectively. One-way ANOVA and t-tests were applied to parametric data. A p-value of less than 0.05 was deemed statistically significant for every analysis.

## Results

### Identification of molecular subtypes of HCC

**Fig. 1:**
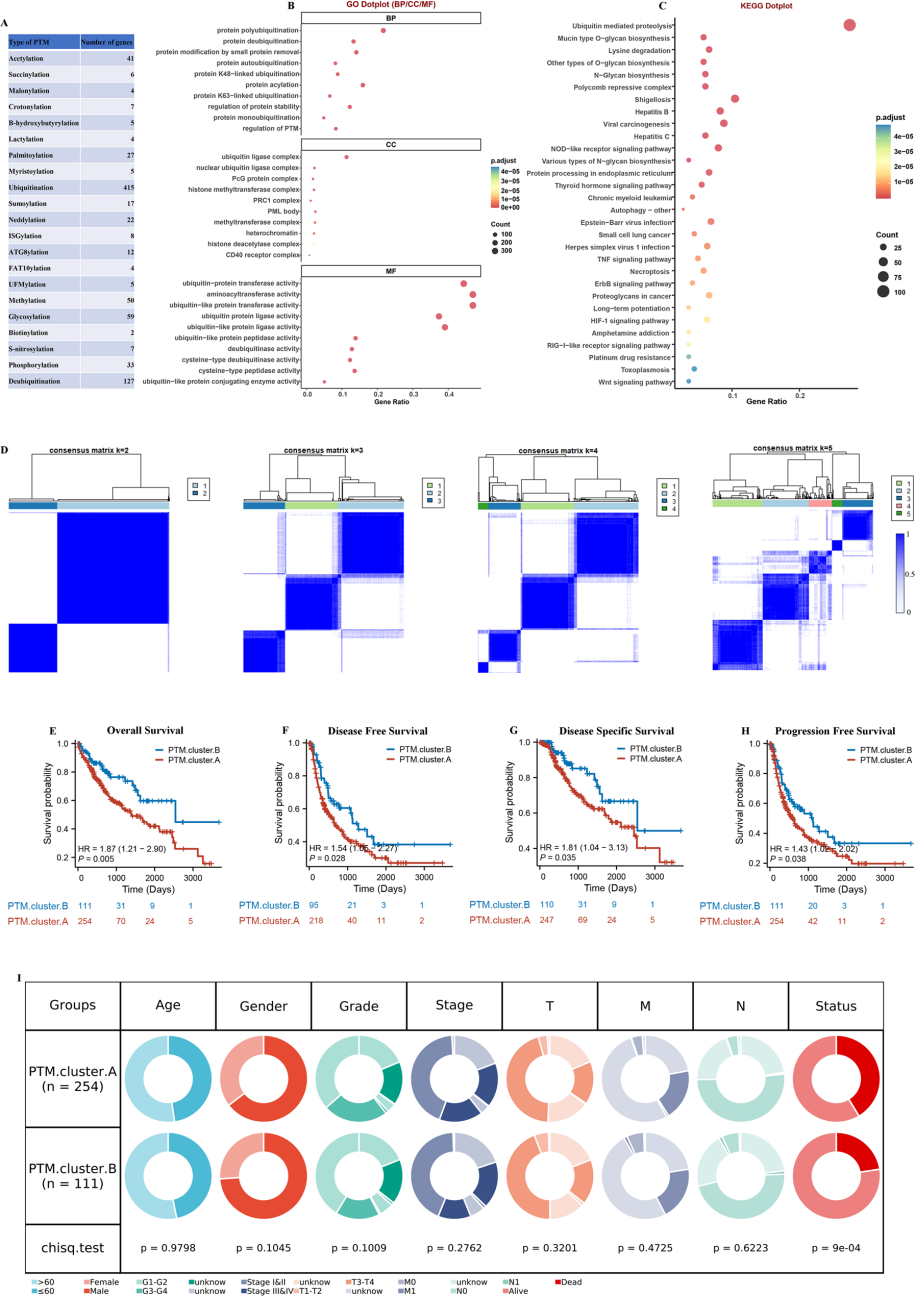
Identification of molecular subtypes of HCC. (**A**) The 21 types of PTMs used in this study. (**B**) GO and (**C**) KEGG enrichment analysis on PTMs. (**D**) The consensus matrix of the clustering analysis. (**E**-**H**) KM curves for the OS, DFS, DSS, PFS of HCC patients among different molecular subtypes. (**I**) Clinicopathological characteristics between two molecular subtypes

In the examination of 21 different types of PTMs, there was a noteworthy variation in the number of associated genes. The gene counts fluctuated significantly, with some PTMs linked to as few as 2 genes, while others were associated with as many as 415 genes, as illustrated in Fig. 1A. This range highlights the diverse genetic landscape related to various PTMs. Subsequently, GO and KEGG analyses were conducted, revealing that the majority of these genes play key roles in the PTM pathways, as well as immune system pathways. The results of these analyses are presented in Fig. 1B and C, underscoring the significant involvement of these genes in critical biological processes related to PTMs and immune responses.

Then, consensus clusters were generated for PTM profiles by employing clustering techniques, which allowed for a detailed analysis of 21 different types of PTMs. The analysis revealed that optimal clustering of the data was achieved when the number of clusters, denoted as k, was set to 2, as illustrated in Fig. 1D and supported by additional evidence in Supplementary Figs. 1 A and 1B. Further examination of survival outcomes indicated that individuals designated to PTM.cluster.A had significantly poorer OS, DSS, DFS, and PFSin comparison to those assigned to PTM.cluster.B (Fig. 1E-H). Subsequently, we conducted a comparison between two distinct subtypes with regard to their clinical characteristics. A significant finding emerged from this comparison: patients classified under the PTM.cluster.A subtype exhibited considerably poorer status when compared to those in the PTM.cluster.B subtype within the TCGA-LIHC dataset (Fig. 1I). This finding suggests that the clustering based on PTMs could serve as a valuable indicator of patient prognosis, highlighting the potential of these modifications in influencing clinical outcomes in HCC.

### Genomic alterations in molecular subtypes of HCC

**Fig. 2:**
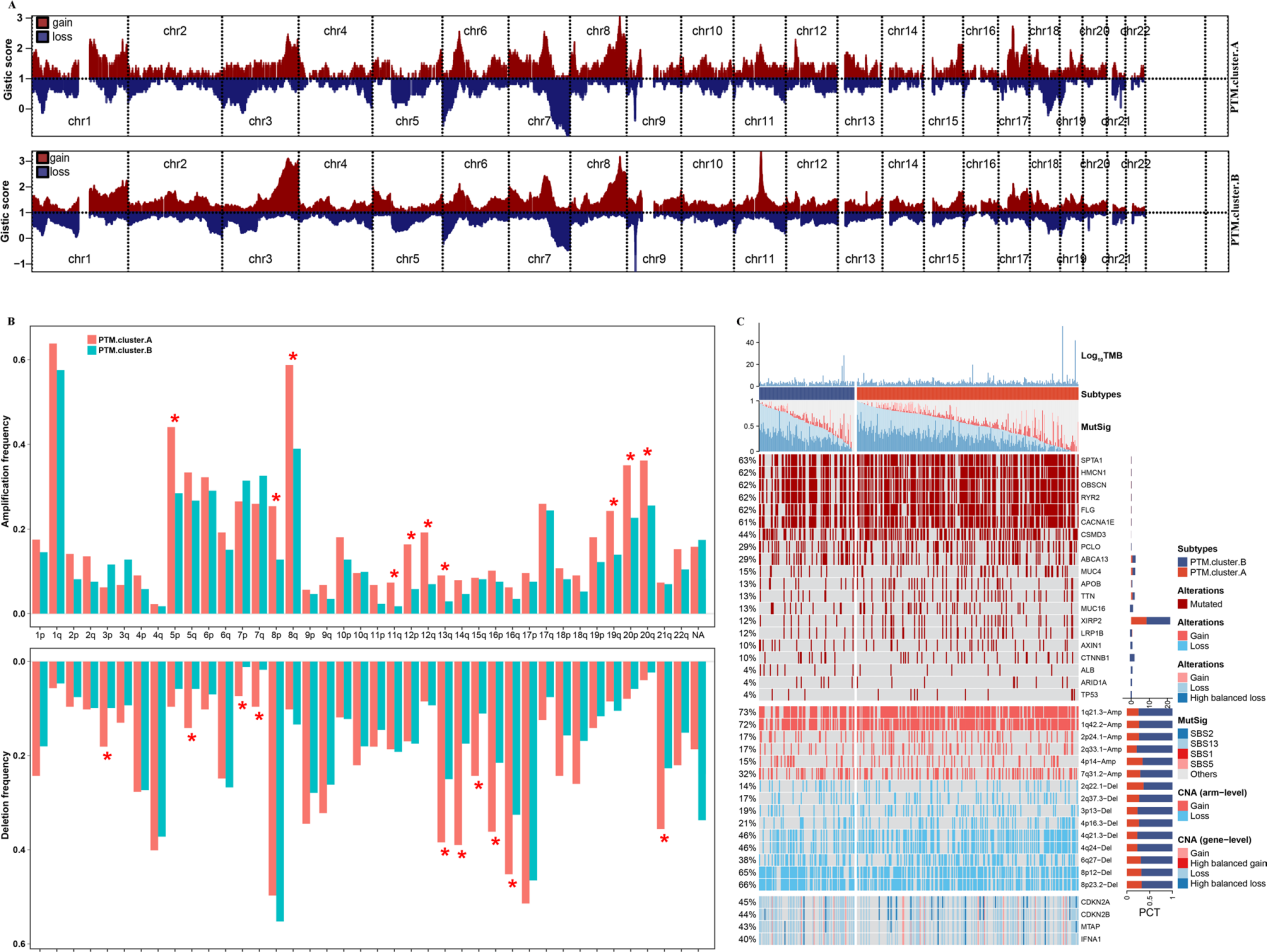
Characteristics of the genomic alterations in molecular subtypes. (**A**) Chromosomal amplifications and deletions between molecular subtypes. (**B**) Mutation information between molecular subtypes. (**C**) The deletion frequency and amplification frequency between molecular subtypes

In the course of our investigation, we conducted a thorough examination of the levels of chromosomal instability, comparing them in relation to molecular subtypes. The findings revealed a significant increase in chromosomal instability among individuals with PTM.cluster.A, especially when contrasted with those in PTM.cluster.B (Fig. 2A). This suggests a strong correlation between elevated molecular subtypes and increased chromosomal instability, highlighting the potential implications of molecular subtypes in understanding chromosomal behavior. Following this analysis, we proceeded to explore the differences in somatic mutations that were linked to the molecular subtypes. The results from this examination indicated that individuals classified in PTM.cluster.A displayed a notably higher frequency of somatic mutations compared to their counterparts in PTM.cluster.B (Fig. 2B). This finding underscores the relationship between elevated molecular subtypes and an increased rate of mutation, which may have significant ramifications in terms of genetic stability and the potential for disease progression (Fig. 2C).

### Immune cell infiltration

**Fig. 3:**
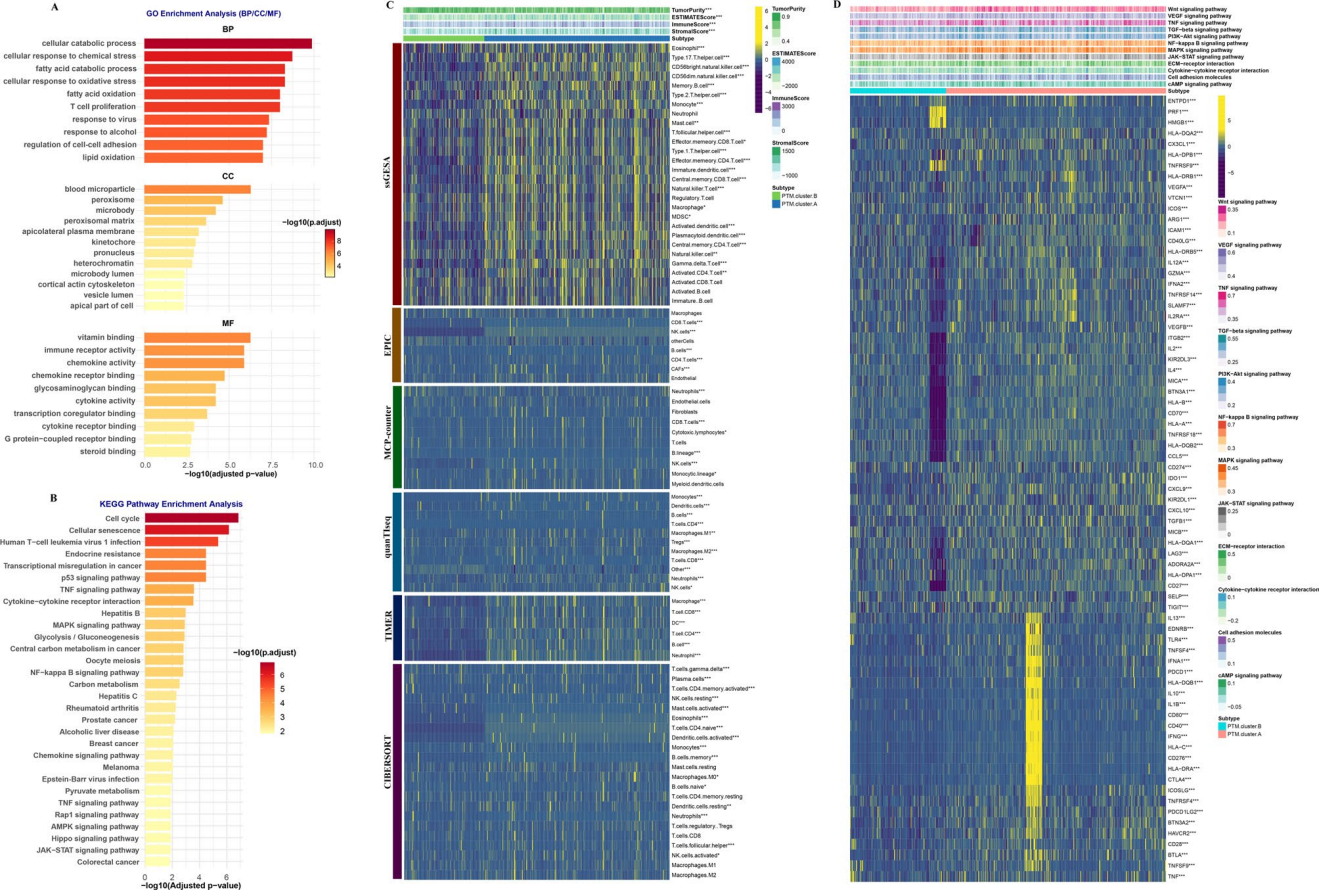
Immune cell infiltration of molecular subtypes. (**A**) GO and (**B**) KEGG analysis of DEGs. (**C**) Immune cell infiltration difference between molecular subtypes. (**D**) The immune modulator molecules expression between molecular subtypes

Then, based on the mRNA expression of PTM-related genes, PCA effectively illustrated the differences between the two identified subtypes (Supplementary Figs. 2). Following the identification of DEGs across the two clusters, a total of 267 shared DEGs were recognized (Supplementary Table 2). Further analysis through GO and KEGG pathways demonstrated that these DEGs were predominantly enriched in various immune pathways (Fig. 3A and B). This suggests that these DEGs might be pivotal in the progression and development of HCC.

Utilizing the ESTIMATE algorithm, we found significant differences in immune metrics between two distinct groups identified as PTM.cluster.A and PTM.cluster.B. Specifically, the PTM.cluster.A group exhibited elevated scores in terms of immune cell infiltration when compared to PTM.cluster.B (Fig. 3C). This finding suggests that the biological environment associated with PTM.cluster.A is more conducive to immune engagement. Furthermore, an in-depth examination of the immune landscape, assessed through the lens of seven distinct algorithms. The analysis indicated that the PTM.cluster.A group contained a richer variety and higher abundance of immune cells compared to the PTM.cluster.B group (Supplementary Figs. 2 A-F). This emphasizes the potential for a more active immune response in individuals within PTM.cluster.A. In addition to the differences in immune cell populations, the levels of immune modulators were also found to be significantly greater in the PTM.cluster.A group than in PTM.cluster.B. This could imply that PTM.cluster.A is enriched with factors that enhance immune activity and regulation, further supporting the notion of a robust immune system in meta cohort (Fig. 3D). Moreover, essential pathways were notably higher in the PTM.cluster.A group compared to those in PTM.cluster.B (Fig. 3D). This heightened pathway activation may play a crucial role in the immune response and could be implicated in the progression of HCC. In summary, the findings suggest that patients belonging to PTM.cluster.A demonstrate a significantly greater degree of immune infiltration, an abundance of immune modulators, and enhanced signaling pathway activity. These characteristics may collectively contribute to the advancement of HCC, indicating a potential area for further research and therapeutic targeting.

### WGCNA analysis

**Fig. 4:**
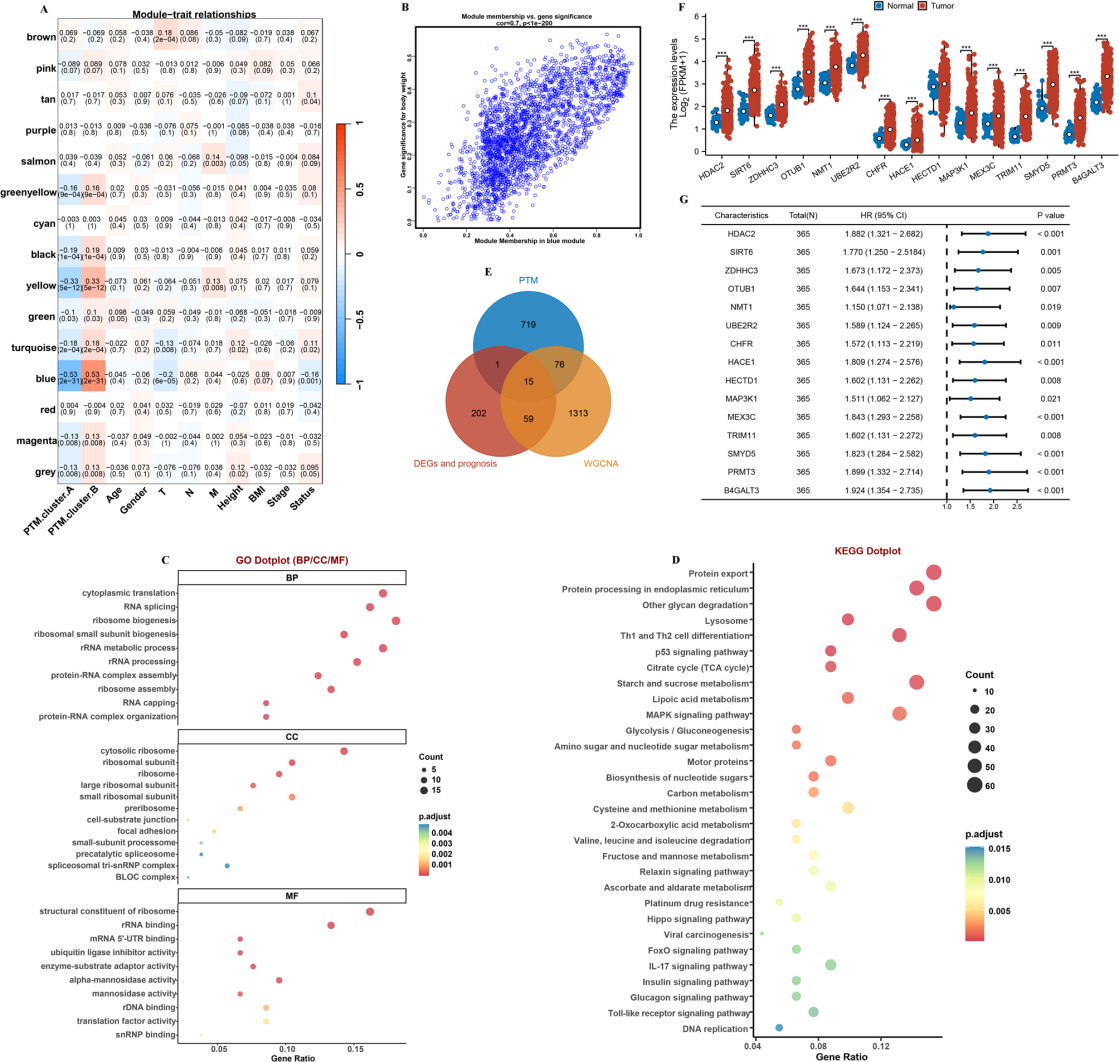
WGCNA analysis. (**A**) Correlation analysis between module eigengenes and clinical traits. (**B**) The correlation between GS and MM in the blue module. (**C**) GO and (**D**) KEGG enrichment analysis on the key module genes. (**E**) Venn diagram showing the 15 marker genes. (**F**) The expression of 15 genes between normal and HCC. (**G**) The OS for the 15 genes

To uncover the significant module genes linked to the progression of HCC, the WGCNA algorithm was utilized. Initially, the Pearson correlation coefficient was computed for each gene across the meta-cohort. The analysis indicated that a β value of 5 yielded a high determination coefficient (R²=0.85), signifying a robust relationship among the genes (Supplementary Figs. 4 A). Subsequently, a dendrogram of gene modules was generated by assessing the differences between these modules, leading to the identification of a total of 15 distinct modules (Supplementary Figs. 4B). The analysis revealed that the blue module exhibited a negative correlation with PTM.cluster.A (*r*=−0.53, *P* < 0.001, Fig. 4A and B). Additionally, GO and KEGG pathway analyses highlighted that the key module genes were predominantly enriched in metabolism-related biological processes. Collectively, these findings suggest that the identified key module genes are likely to play significant roles in the development of HCC. Finally, a specific selection was made of the 15 overlapping genes found among the DEGs and prognosis genes identified between the two subtypes and the key module genes and PTM genes (Fig. 4E and G). These genes will be the focus of further investigation to enhance our understanding of their contributions to the HCC.

### Construction of ptm.score

**Fig. 5:**
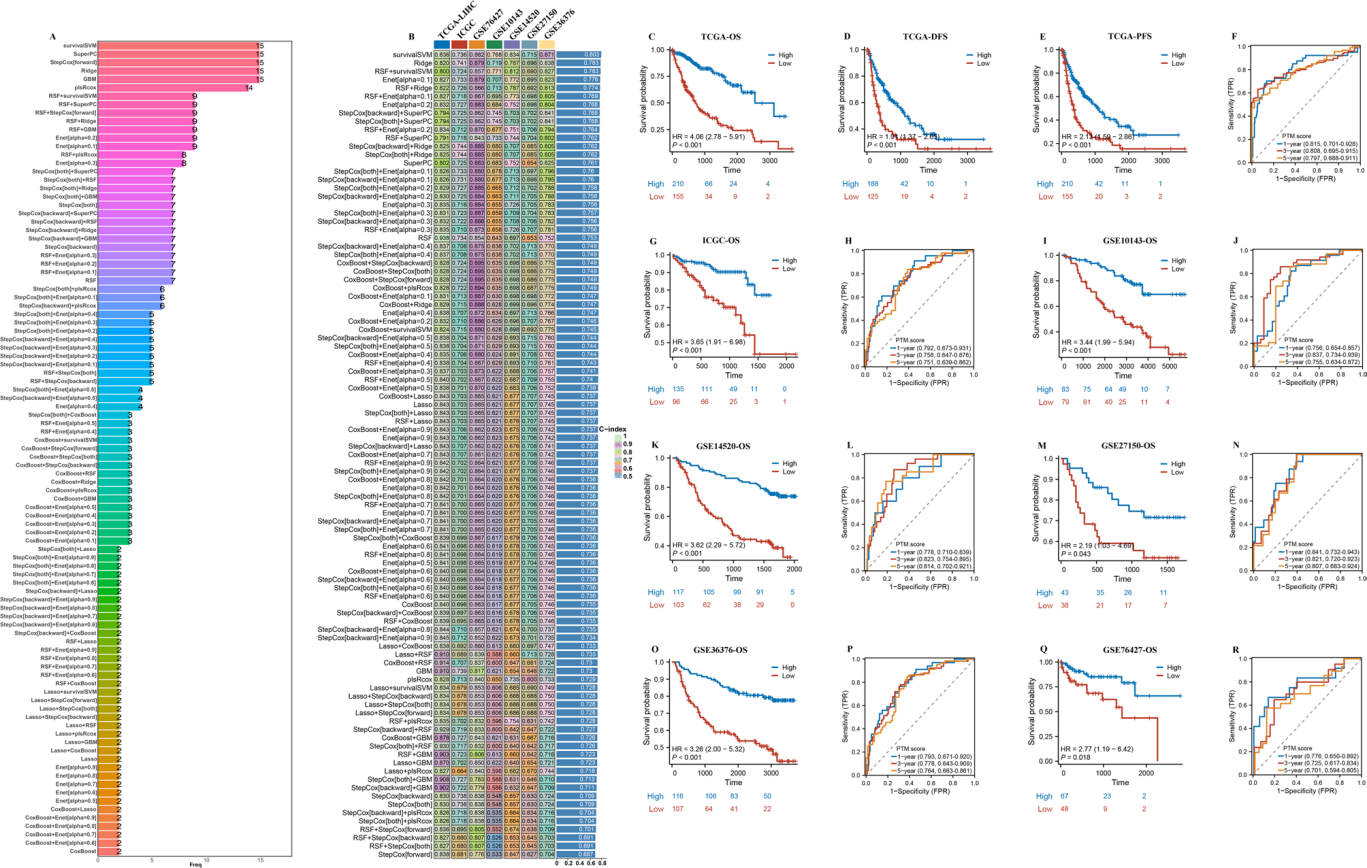
Construction of PTM.score. (**A**) The most valuable overlapping genes based on the multiple algorithms. (**B**) Hub genes were identified by machine learning algorithms. KM curves for the OS (TCGA-LIHC) (**C**), DFS (TCGA-LIHC) (**D**), PFS (TCGA-LIHC) (**E**), OS (ICGC) (**G**), OS (GSE10143) (**I**), OS (GSE14520) (**K**), OS (GSE27150) (**M**), OS (GSE36376) (**O**), OS (GSE76427) (**Q**) of HCC patients among high/low PTM.score. Time-dependent ROC curves of 1-year, 3-year, and 5-year OS in the TCGA-LIHC (**F**), ICGC (**H**), GSE10143 (**J**), GSE14520 (**L**), GSE27150 (**N**), GSE36376 (**P**), GSE76427 (**R**)

It was particularly fascinating to note that the primary model identified in our analysis was survivalSVM. This hybrid model exhibited remarkable performance metrics, attaining an outstanding average C-index of 0.803. This notable result not only highlights the model’s effectiveness in assessing risks but also reinforces its reliability as a tool for such evaluations. The high C-index value indicates that the model has a strong capacity to accurately predict outcomes, making it an important asset in the field of risk assessment (Supplementary Figs. 5, Supplementary Figs. 6 A, Fig. 5A and B). Further examination of survival outcomes indicated that individuals designated to low PTM.score had significantly poorer OS (TCGA-LIHC, ICGC, GSE10143, GSE14520, GSE27150, GSE36376, and GSE76427), DFS (TCGA-LIHC), and PFS (TCGA-LIHC) in comparison to those assigned to high PTM.score. Then, AUC values for the PTM.score at the 1-year, 3-year, and 5-year marks in the TCGA-LIHC were significantly high, with values recorded at 0.815, 0.808, and 0.797, 0.792, 0.758, and 0.751 in the ICGC, 0.756, 0.837, and 0.755 in the GSE10143, 0.778, 0.823, and 0.814 in the GSE14520, 0.841, 0.821, and 0.807 in the GSE27150, 0.793, 0.778, and 0.764 in the GSE36376, and 0.776, 0.725, and 0.701 in the GSE76427 (Fig. 5C and R). These results indicate a strong predictive capability of the PTM.score over the specified time intervals, underscoring its potential utility in clinical assessment and decision-making processes.

### Evaluation of Ptm.score

**Fig. 6:**
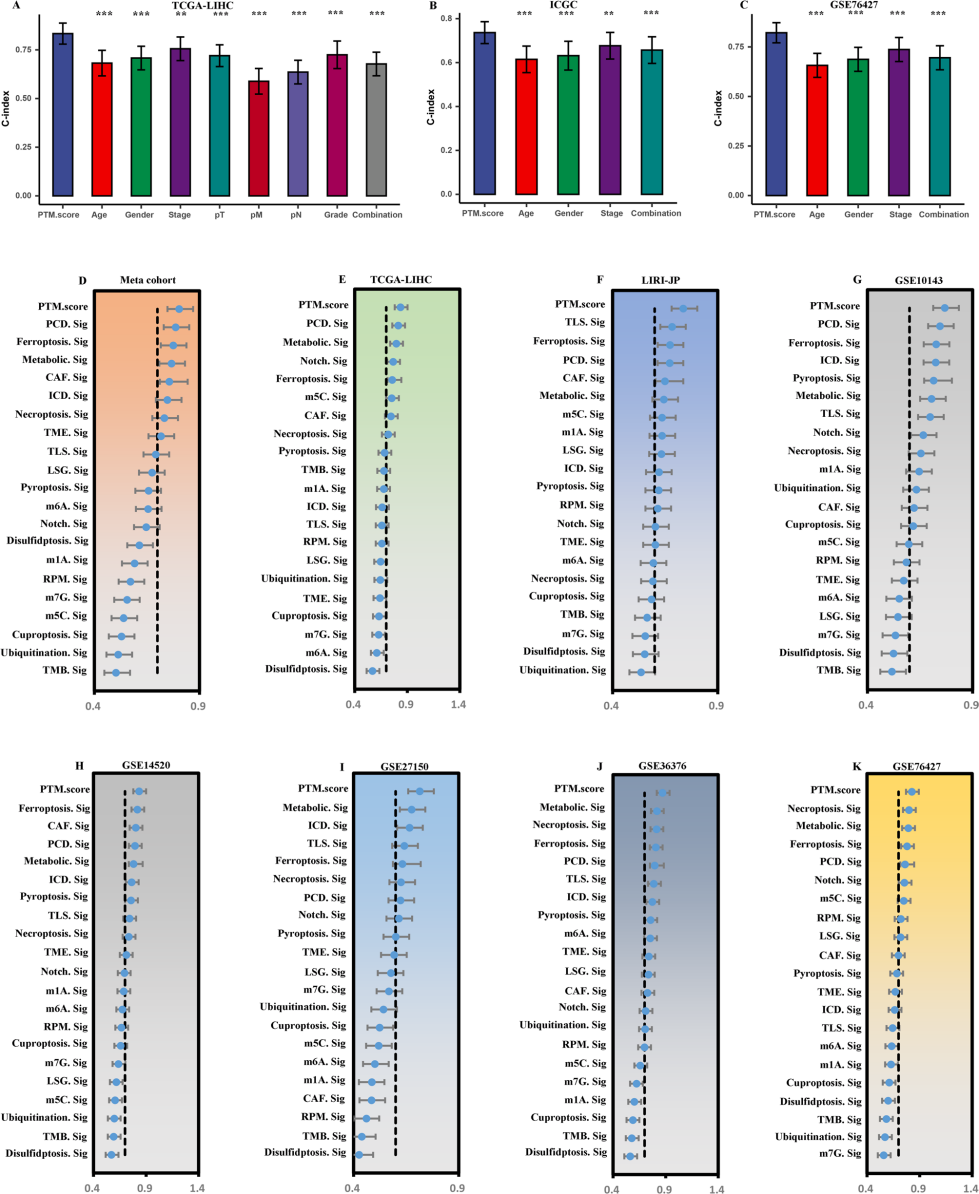
Model evaluation of PTM.score. (**A**-**C**) Comparison of PTM.score with other clinical features in TCGA-LIHC, LIRI-JP, and GSE76427. (**D**-**K**) Comparative analysis with 20 published signatures in meta-cohort, TCGA-LIHC, LIRI-JP, GSE10143, GSE14520, GSE27150, GSE36376, and GSE76427

The subsequent analysis demonstrated that the PTM.score showed notably superior C-index values in comparison to all other clinical features that were evaluated. These features encompassed a range of factors, highlighting the enhanced predictive capability of the PTM.score over traditional clinical metrics (Supplementary Figs. 6B, Fig. 6A and C). The results of our analysis suggest that the PTM.score demonstrates a greater level of reliability as a predictive tool in clinical settings when compared to traditional indicators. To further investigate its efficacy, we performed a comparative analysis of the PTM.score against a set of 20 previously validated signatures for HCC. The outcomes of this comparison were noteworthy, as the PTM.score consistently outperformed all other signatures across six different datasets, achieving the highest C-index values among those assessed (Fig. 6D and K). The impressive performance demonstrated by PTM.score underscores its considerable value in effectively predicting patient outcomes. This capability not only enhances its relevance in clinical settings but also solidifies its role as a vital tool in research focused on oncology. The ability to accurately assess patient prognosis is crucial, as it can influence treatment decisions and improve overall patient care. Therefore, the significance of PTM.score extends beyond mere statistics; it represents a meaningful advancement in the landscape of cancer treatment and research.

**Fig. 7:**
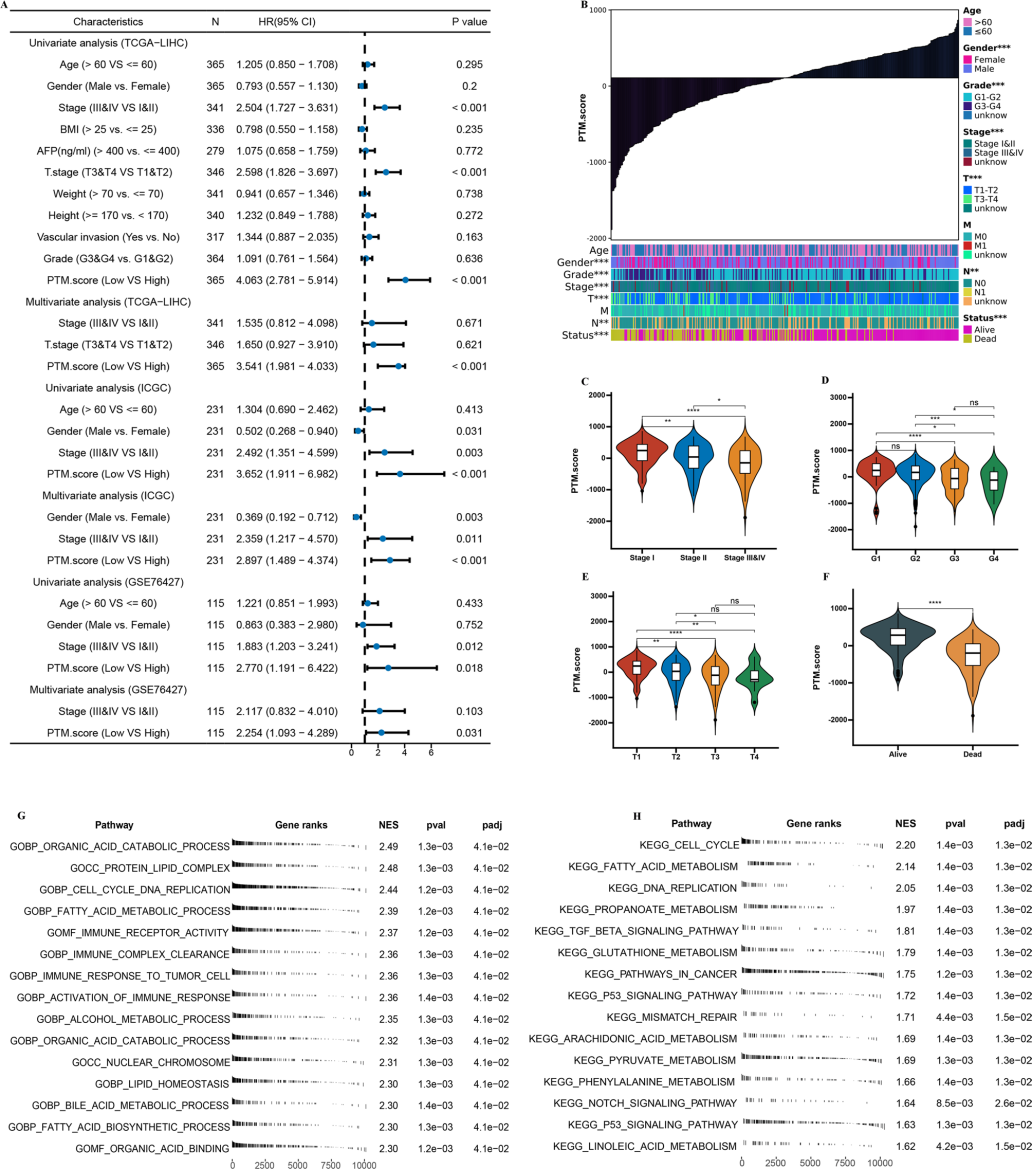
Characteristics of PTM.score. (**A**) Univariate and multivariate Cox regression analyses of PTM.score. (**B**-**F**) Analysis of differences in PTM.score among clinicopathological characteristics. (**G**) GSEA GO and (**H**) GSEA KEGG enrichment analysis on PTM.score

Then, we utilized a combination of univariate and multivariate Cox regression analyses. This result underscores the robustness of the PTM.score as a valuable prognostic tool, indicating that its relevance remains strong regardless of other clinical parameters that may typically affect patient outcomes. Such findings emphasize the critical role of the PTM.score in clinical practice, as it has the potential to inform treatment decisions and enhance patient management strategies based on its predictive efficacy (Fig. 7A). The results also indicated that patients classified high stage exhibited lower PTM.score (Fig. 7B and F). Subsequently, we employed GSEA to identify signaling pathways associated with elevated PTM scores. The results, illustrated in Fig. 7G and H, indicate a significant enrichment of pathways linked to immune responses in samples exhibiting low PTM scores.

### Immunotherapy response to Ptm.score

Utilizing 7 algorithms, we found significant differences in immune metrics between high and low PTM.score groups. Specifically, the low PTM.score exhibited elevated scores in terms of immune cell infiltration when compared to high PTM.score (Supplementary Figs. 7). This finding suggests that the biological environment associated with high PTM.score is more conducive to immune engagement.

**Fig. 8:**
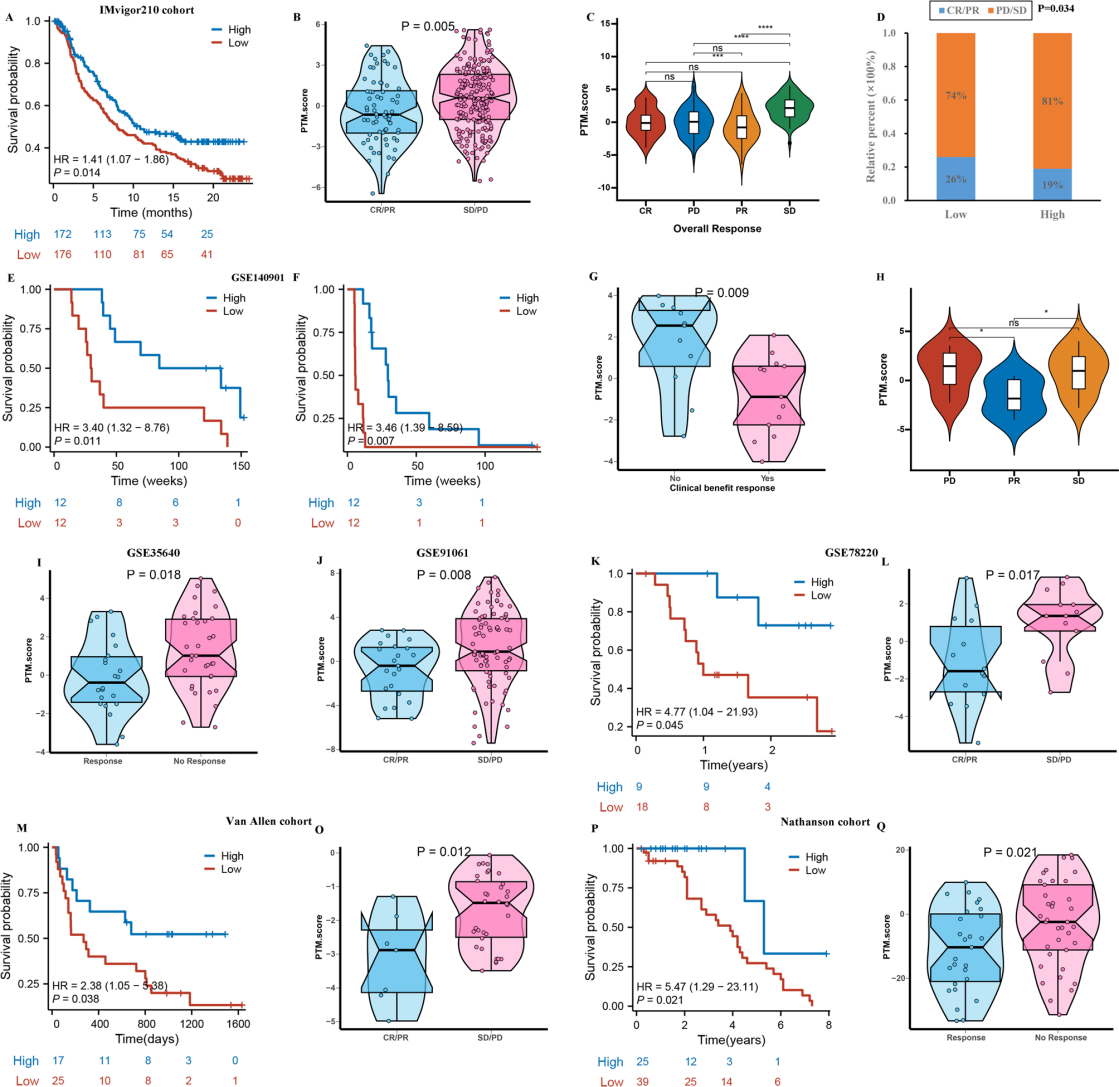
Immunotherapy response to PTM.score. (**A**) Survival analyses for high/low PTM.score in IMvigor210 cohort. (**B**-**C**) Differences in PTM.score among distinct anti-PD-1 clinical response groups. (**D**) The proportion of patients with response to PD-L1 blockade immunotherapy in high/low PTM.score. Survival analyses for high/low PTM.score in GSE140901 (**E**, **F**), GSE78220 (**K**), Van Allen cohort (**M**), and Nathanson dataset (**P**). Box plot displaying the PTM.score in patients with different immunotherapy responses in the GSE140901 (**G**, **H**), GSE35640 (**I**), GSE91061(**J**), GSE78220 (**L**), Van Allen cohort (**O**), and Nathanson dataset (**Q**)

Then, individuals with low PTM scores demonstrated poorer OS rates in comparison to their counterparts with high PTM.score (Fig. 8A). Additionally, the PTM.score for the SD/PD group was found to be higher than that of the CR/PR group within the IMvigor cohort (Fig. 8B and C). Furthermore, the CR/PR was significantly lower in the group with high PTM.score compared to those with low PTM.score, with figures standing at 19% and 26%, respectively (Fig. 8D). Subsequently, low PTM.score exhibited a significantly reduced lifespan in contrast to those with high PTM.score in Van Allen, Nathanson, GSE35640, GSE78220, GSE91061, GSE140901. Furthermore, the findings demonstrated a significant increase in the PTM.score within the SD/PD groups in an Allen, Nathanson, GSE35640, GSE78220, GSE91061, GSE140901 (Fig. 8E and Q). This comprehensive analysis underscores the variability in immunotherapy response associated with PTM.score across different databases, highlighting the importance of personalized treatment strategies based on individual patient profiles.

### Validation of Ptm.score

**Fig. 9:**
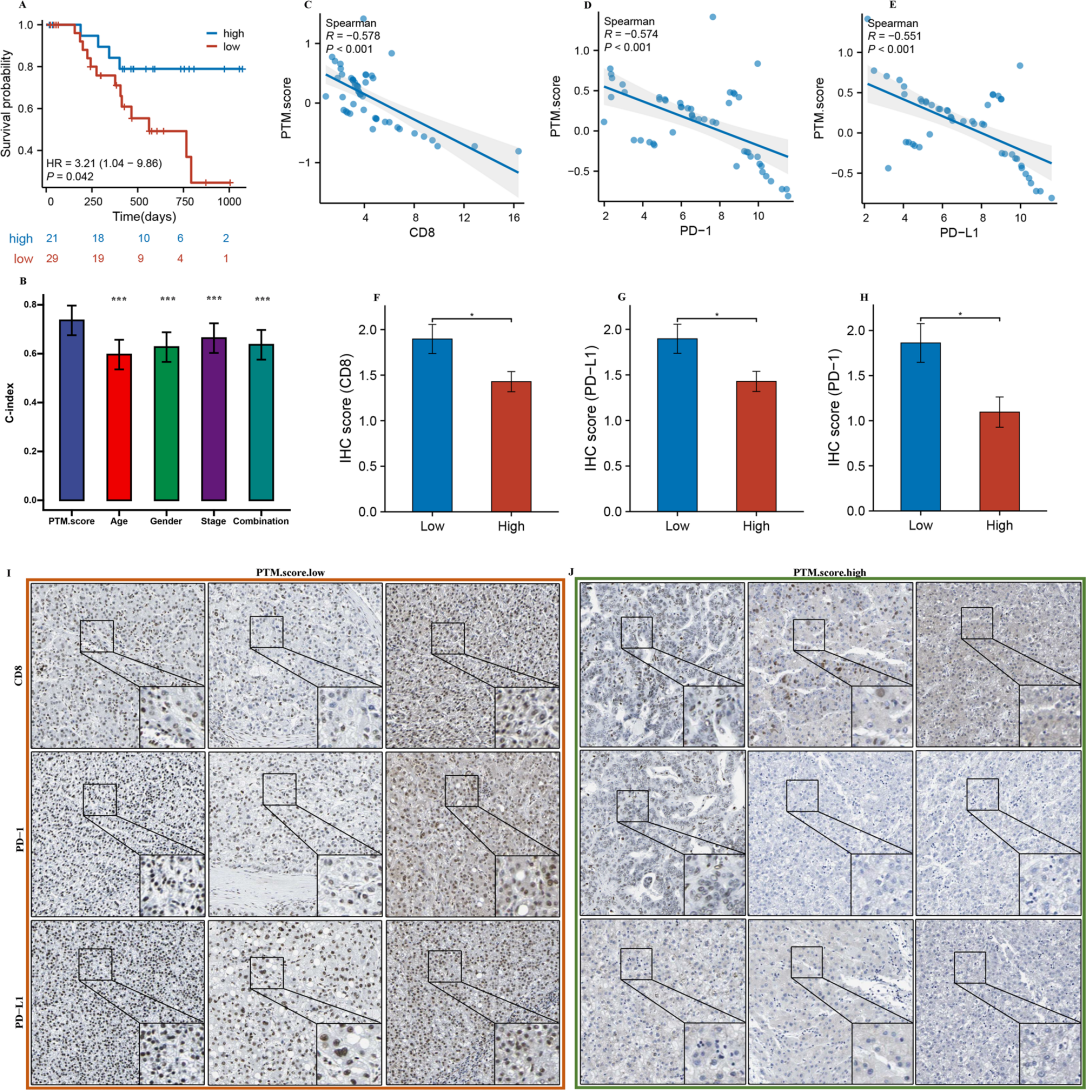
Validation of PTM.score in house cohort. (**A**) Different OS outcomes among the high/low PTM.score groups. (**B**) Comparison of PTM.score with other clinical features in house cohort. (**C**-**E**) Scatter plot displaying the correlation between the PTM.score and CD8, PD-1, and PD-L1. (**F**-**J**) Representative IHC staining images of CD8, PD-1, and PD-L1 in high/low PTM.score groups

Further examination of survival outcomes indicated that individuals designated to low PTM.score had significantly poorer OS in comparison to those assigned to high PTM.score, and PTM.score showed notably superior C-index values in comparison to all other clinical features in the house cohort (Fig. 9A and B). The results demonstrated a negative correlation with PTM.score and the expression of CD8A, PD-1, and PD-L1 (Fig. 11C and E). Furthermore, immunohistochemical analyses further supported these findings by demonstrating that the group with low PTM.score showed significantly increased levels of CD8A, PD-1, and PD-L1 when compared to the group with a high PTM.score (Fig. 9F and J). This suggests that individuals within the low PTM.score category are likely experiencing enhanced immune activation, as reflected by the increased expression of these critical immune markers.

#### Expression pattern of Ptm.score in scRNA-seq data

**Fig. 10:**
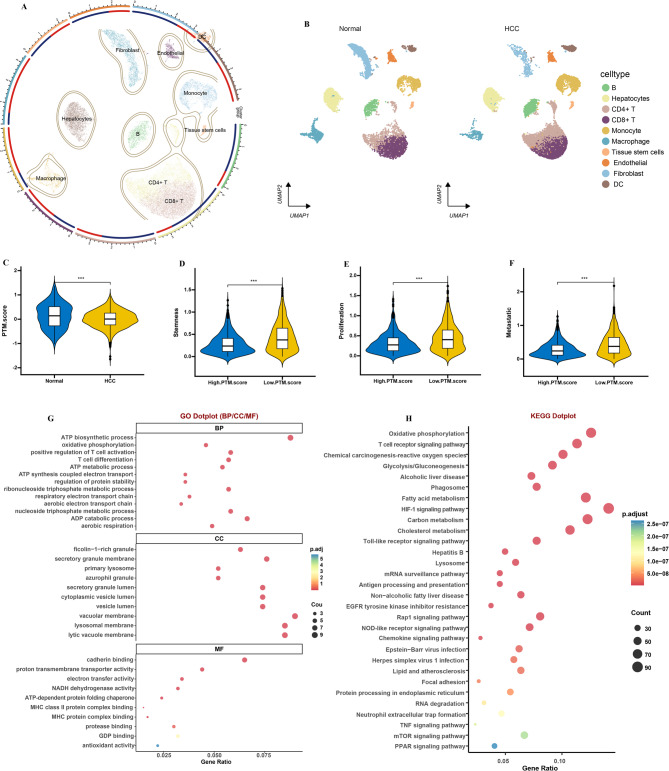
Single-cell analysis of PTM.score. (**A**, **B**) 10 cell types were identified by performing UMAP. (**C**) Illustrating the differences in PTM.score between the normal and HCC groups. Violin plots displaying the expression scores of stemness (**D**), proliferation (**E**), and metastatic (**F**) in tumor cells from the high PTM.score and low PTM.score groups. (**G**) GO and (**H**) KEGG analysis of DEGs

This analysis was confined to HCC data from five healthy donors and five HCC patients, sourced from the GSE242889 dataset. The scRNA-seq quality-control workflow (UMI counts, mitochondrial gene proportion, doublet rate) and marker genes used for cell-type annotation were showed in Supplementary Figs. 8 A-D. Cell-type classification was conducted utilizing the SingleR package, which categorized the clusters into 10 primary immune cell types based on the expression of established marker genes (Fig. 10A and C). HCC patients showed notably superior PTM.score in comparison to healthy donors (Fig. 10D). Moreover, the tumor cells characterized by a low PTM.score demonstrated significantly elevated expression levels of markers associated with stemness, proliferation, and metastasis when compared to their counterparts in the high PTM.score group (Supplementary Table 4). This observation indicates that tumors with lower PTM.scores may exhibit a more aggressive and malignant phenotype, thereby suggesting a correlation between low PTM.scores and increased tumor malignancy (Fig. 10D and F). Subsequently, GO and KEGG analyses were conducted, revealing that the majority of DEGs between high/low PTM.score group play key roles in the ATP metabolism pathways, as well as immune system pathways (Fig. 10G and H).

### Expression pattern of Ptm.score in spatial transcriptomics

**Fig. 11:**
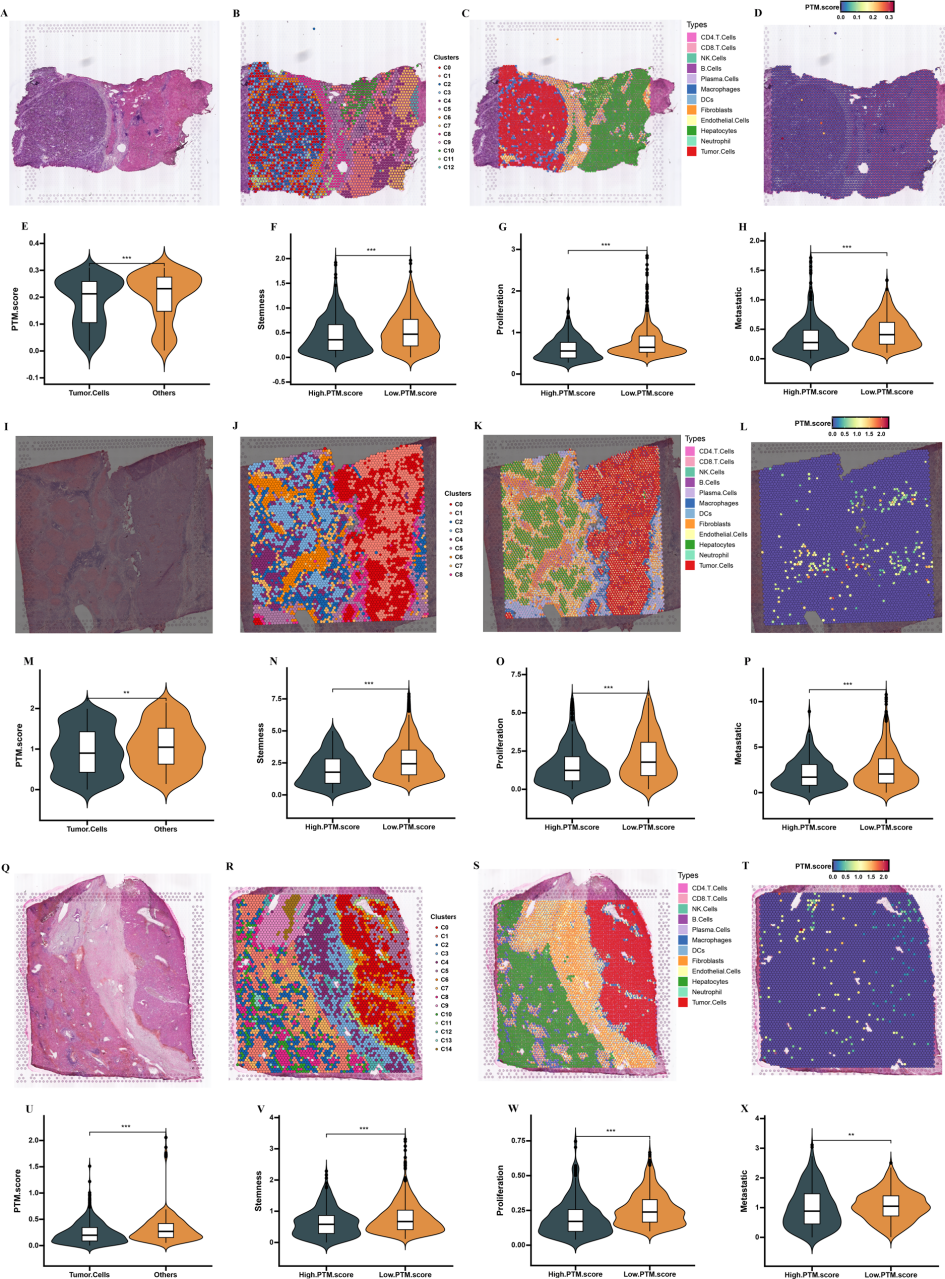
Spatial transcriptomics analysis of PTM.score**. **Representative H&E staining of tissue sections of HCC-1L (**A**), HCC-2L (I), and HCC-3L(**Q**). Cell clusters were identified by performing UMAP of HCC-1L (**B**), HCC-2L (**J**), and HCC-3L(**R**). Cell types were identified by performing UMAP of HCC-1L (**C**), HCC-2L (**K**), and HCC-3L(**S**). The relative abundance of PTM.score in cell types of HCC-1L (**D**), HCC-2L (**L**), and HCC-3L(**T**). Illustrating the differences in PTM.score between the tumor cells and other cells in HCC-1L (**E**), HCC-2L (**M**), and HCC-3L (**U**). Violin plots displaying the expression scores of stemness in HCC-1L (**F**), HCC-2L (**N**), and HCC-3L (**V**), proliferation in HCC-1L (**G**), HCC-2L (**O**), and HCC-3L (**W**), and metastatic in HCC-1L (**H**), HCC-2L (**P**), and HCC-3L (**X**) in tumor cells from the high PTM.score and low PTM.score groups

To further elucidate the expression patterns of PTM.score, HRA000437 (HCC-1 L, HCC-2 L, and HCC-3 L) were utilized to investigate the spatial distribution and expression characteristics of PTM.score. The spatial transcriptomics data obtained from three tissue samples were carefully chosen for further analysis through a detailed microscopic examination of H&E-stained sections (Fig. 11A, I and Q). In the processing of spatial transcriptomics data, the information from each individual was handled separately to ensure accuracy and specificity in the analysis. Following the normalization of gene expression data and the implementation of PCA to assess genes that were variably expressed, the data points referred to as spots were then analyzed through unsupervised clustering methods. This analysis revealed a total of 12 clusters on HCC-1 L, 8 clusters on HCC-2 L, and 14 clusters on HCC-3 L, illustrating the complexity and heterogeneity within the tissue samples examined (Fig. 11B, J and R). Subsequently, we illustrated the occupation and enrichment of cell types through feature plots, which were generated by scoring the spots according to cell type-specific signatures. This method provided a clearer visualization of the distribution and prevalence of each cell type within the defined clusters (Fig. 11C, K and S). Spatial transcriptomic analysis revealed distinct PTM.score profiles localization in tissues (Fig. 11D, L and T). The results indicate that tumor cells showed notably lower PTM.score in comparison to other cells (Fig. 11E, M and U). Moreover, tumor cells characterized by a low PTM.score demonstrated significantly elevated levels of expression related to stemness, proliferation, and metastatic traits when compared to their counterparts in the high PTM.score group. This observation indicates that tumors within the low PTM.score category tend to possess a greater degree of malignancy, reflecting their potentially more aggressive nature. (Figure 11F, H, N, P, V and X).

## Discussion

HCC stands as one of the most common cancers worldwide, prompting significant research into its mechanisms of development and potential immunotherapy treatments. PTMs are critical in the progression of HCC. For example, protein acetylation participates in tumor cell migration related to tumor angiogenesis. SPZ1 acts as a proto-oncogene and forms an SPZ1-TWIST1 complex through the acetylation of TWIST1 during tumorigenesis, which is essential for cancer cell migration [35]. Moreover, MRPS5 has been shown to play an important role in the metabolic reprogramming of hepatocellular carcinoma cells. Bioinformatic analysis indicated that MRPS5 is closely related to the function of mitochondrial complex I. Further experiments confirmed that MRPS5 promoted the production of NAD, enhanced the mitochondrial of hepatocellular carcinoma, and participated in energy regulation. Acetylation of MRPS5 could directly lead to an increase in the expression of glycolytic proteins promote the Warburg effect. In contrast, deacetylation of MRPS5, which is localized in the mitochondria, promoted the function of mitochondrial complex-I and production of NAD, enhanced mitochondrial respiration, and regulated the energy metabolism of cancer cells [36]. This emerging evidence highlights the important role that PTMs play in cancer biology, particularly in the context of enhancing treatment responses. The findings from studies investigating PTMs across different cancer types yield important insights into their functional ramifications in HCC. Our thorough examination of PTMs has revealed their critical participation in the development and advancement of HCC. By aggregating data from a substantial pool of 1,398 patients diagnosed with HCC from four distinct international cohorts, we have developed a novel computational tool known as PTM.score. This framework proficiently captures and reflects the prognostic significance of 15 vital PTMs, thus contributing to a more nuanced understanding of their impact on patient outcomes in HCC. The applicability of this model extends beyond HCC itself, having been validated across 7 immunotherapy cohorts, including melanoma, HCC, and urothelial cancer. Importantly, PTM.score has demonstrated superior prognostic accuracy when compared to 20 existing HCC signatures. This positions PTM.score as an invaluable tool for personalizing immunotherapy strategies, thereby enhancing the optimization of patient outcomes through more tailored treatment approaches.

In terms of immune evasion, cancer cells employ various mechanisms to avoid detection and elimination by the host immune system, such as upregulating the expression of immune checkpoint proteins to suppress T cell activation and secreting immune inhibitory factors to create an immunosuppressive microenvironment [37]. Additionally, tumors promote their growth by triggering chronic inflammatory responses. Research teams have discovered that in liver cancer, pro-inflammatory factors released by hepatocytes drive macrophages toward a pro-inflammatory phenotype, exacerbating liver inflammation and promoting liver cancer growth [38]. Finally, in terms of metabolic reprogramming, cancer cells and immune cells compete for nutrients and oxygen in the TME, affecting immune cell function and cancer cell survival [39]. For example, cancer cells deplete glucose in the local environment through efficient glycolysis, thereby reducing T cell activity. Therefore, a thorough understanding of these characteristics related to TME is crucial for developing innovative cancer treatment regimens and improving the effectiveness of existing treatments. In the process of cancer development, anti-tumor T cells are unable to control tumor growth due to tumor-induced tolerance mechanisms. A typical manifestation of this dysfunction is the marked expression of inhibitory receptors on the surface of T cells, including CTLA-4 and PD-1 [40]. Along with increased receptor expression, T cell effector function is also significantly impaired, characterized by reduced ability to produce key cytokines such as IFNγ, IL-2, and TNFα [41]. Additionally, a significant reduction in T cell proliferative potential exacerbates this impaired state. The reversible and irreversible states of T cell exhaustion have been confirmed, and irreversibly exhausted cells do not respond to ICB-type anti-PD-1/anti-PD-L1 therapy [42]. This is consistent with our results, patients categorized with a PTM.cluster.A displayed a TME that was more actively engaged, which corresponded with a poor prognosis. Next, we performed functional analysis on the two subgroups to explore their potential biological mechanisms. Based on the differentially expressed genes identified, GO analysis and KEGG analysis indicated that immune dysregulation may mediate the role of PTM in the occurrence and development of HCC.

In the treatment of recurrent and metastatic HCC, clinical studies have established the status of ICIs, which have also the foundation for the subsequent exploration of the synergistic effect of radiotherapy combined with immunotherapy. Although immunotherapy monotherapy shows survival benefits, there is room for improvement in response rates. At present, pembrolizumab is approved as an immunotherapy drug for the first-line treatment of recurrent and metastatic HCC [43]. In one, the degree of benefit from pembrolizumab treatment was stratified by the CPS, but the objective response rate (ORR) was about 10%~20% in HCC patients treated [44]. The variability observed in cancer treatment outcomes underscores the intricate nature of managing this disease and emphasizes the urgent need for personalized treatment strategies. The response to ICIs can differ significantly among patients, which indicates that a one-size-fits-all approach is often insufficient. To improve the prediction of how effective these therapies will be for individual patients, researchers are increasingly turning to a range of essential biomarkers. These biomarkers can provide critical insights into a patient’s unique tumor characteristics and immune response, thereby allowing for a more tailored and effective treatment plan. This research demonstrated a significant reduction in the PTM.score within the response groups when evaluated across different cohorts. This advancement in predictive capability has the potential to lead to improved therapeutic outcomes for patients, ultimately enhancing the effectiveness of treatment strategies.

Our findings should be interpreted in light of several limitations. By incorporating PTM into the prognostic model, we effectively capture the complex biological heterogeneity of HCC. This enhances the model’s accuracy and generalizability, offering a more nuanced understanding of patient prognosis. However, the study’s reliance on data from public database may limit the external validity of the model. This constraint could impact the broader applicability of the findings, and further validation in multicenter cohorts is warranted. Finally, the complexity of HCC pathogenesis necessitates a more comprehensive analysis of the interactions between PTM and other genes/proteins involved in the disease.

## Conclusions

In summary, the gene signature associated with novel PTM serves as a valuable prognostic tool for patients with HCC. Our study may offer valuable insights into more scientific treatment strategies for HCC patients. Additionally, we have created an easy-to-use website to facilitate clinical application. Remarkably, PTM.score is a promising target for treatment and may be a significant factor in aggravating the malignant biological behavior of HCC.

## Supplementary Information

Supplementary Material 1.

Supplementary Material 2: 21 Types of post-translational modification gene sets

Supplementary Material 3: DEGs between subtypes

Supplementary Material 4: Retrieved 20 published signatures for HCC

Supplementary Material 5: Cancer stemness and proliferation signatures using in this study

Supplementary Material 6: (A)The CDF curves of consensus matrix for each k. (B) The delta area of consensus matrix for each k. 

Supplementary Material 7: PCA analysis

Supplementary Material 8: The different immune infiltration between the molecular subtypes by using CIBERSORT-ABS (A), EPIC (B), MCPcounter (C), quanTIseq (D), ssGSEA (E), TIMER (F) (*p<0.05，**p<0.01，***p< 0.001).

Supplementary Material 9: (A) The sample similarity of each subgroup was assessed by calculating the Silhoutte score. (B) Identification of co-expression gene modules

Supplementary Material 10: The cross-validation details of machine learning

Supplementary Material 11: (A) LASSO analysis. (B) Collinearity analysis of risk factors

Supplementary Material 12: Immune cell infiltration of high PTM.score and low PTM.score groups

Supplementary Material 13: Analysis of single-cell RNA sequencing data. (A, B) Violin diagram showed the threshold to filter cells. (C) Clustree showed the distinct clusters. (D) Dotplot of marker genes for cell types

## Data Availability

The datasets generated for this study can be found in the GEO database (GSE10143, GSE14520, GSE27150, GSE36376, and GSE76427, GSE91061, GSE78220, Van Allen, and Nathanson; https://www.ncbi.nlm.nih.gov/geo/), and UCSC Xena website (https://gdc.xenahubs.net).

## References

[1] Siegel RL, Kratzer TB, Giaquinto AN, Sung H, Jemal A. Cancer statistics, 2025. Cancer J Clin. 2025;75(1):10–45. 10.3322/caac.21871 PMC11745215 39817679

[2] Wagle NS, Nogueira L, Devasia TP, Mariotto AB, Yabroff KR, Islami F, et al. Cancer treatment and survivorship statistics, 2025. CA Cancer J Clin. 2025;75(4):308–40. 40445120 10.3322/caac.70011 PMC12223361

[3] Shen KY, Zhu Y, Xie SZ, Qin LX. Immunosuppressive tumor microenvironment and immunotherapy of hepatocellular carcinoma: current status and prospectives. J Hematol Oncol. 2024;17(1):25. 38679698 10.1186/s13045-024-01549-2 PMC11057182

[4] Finn RS, Ryoo BY, Merle P, Kudo M, Bouattour M, Lim HY, et al. Pembrolizumab as Second-Line therapy in patients with advanced hepatocellular carcinoma in KEYNOTE-240: A randomized, Double-Blind, phase III trial. J Clin Oncol. 2020;38(3):193–202. 31790344 10.1200/JCO.19.01307

[5] Yau T, Kang YK, Kim TY, El-Khoueiry AB, Santoro A, Sangro B, Melero I, Kudo M, Hou MM, Matilla A, et al. Efficacy and safety of nivolumab plus ipilimumab in patients with advanced hepatocellular carcinoma previously treated with sorafenib: the checkmate 040 randomized clinical trial. JAMA Oncol. 2020;6(11):e204564. 33001135 10.1001/jamaoncol.2020.4564 PMC7530824

[6] Melero I, Yau T, Kang YK, Kim TY, Santoro A, Sangro B, et al. Nivolumab plus ipilimumab combination therapy in patients with advanced hepatocellular carcinoma previously treated with sorafenib: 5-year results from checkmate 040. Ann Oncol. 2024;35(6):537–48. 38844309 10.1016/j.annonc.2024.03.005

[7] Hermann J, Schurgers L, Jankowski V. Identification and characterization of post-translational modifications: clinical implications. Mol Aspects Med. 2022;86:101066. 35033366 10.1016/j.mam.2022.101066

[8] Sharma C, Hamza A, Boyle E, Donu D, Cen Y. Post-translational modifications and diabetes. Biomolecules. 2024;14(3):310. 38540730 10.3390/biom14030310 PMC10968569

[9] Patwardhan A, Cheng N, Trejo J. Post-translational modifications of G protein-coupled receptors control cellular signaling dynamics in space and time. Pharmacol Rev. 2021;73(1):120–51. 33268549 10.1124/pharmrev.120.000082 PMC7736832

[10] Gupta R, Sahu M, Srivastava D, Tiwari S, Ambasta RK, Kumar P. Post-translational modifications: regulators of neurodegenerative proteinopathies. Ageing Res Rev. 2021;68:101336. 33775891 10.1016/j.arr.2021.101336

[11] Wu H, Huang H, Zhao Y. Interplay between metabolic reprogramming and post-translational modifications: from glycolysis to lactylation. Front Immunol. 2023;14:1211221. 37457701 10.3389/fimmu.2023.1211221 PMC10338923

[12] Wang YW, Zuo JC, Chen C, Li XH. Post-translational modifications and immune responses in liver cancer. Front Immunol. 2023;14:1230465. 37609076 10.3389/fimmu.2023.1230465 PMC10441662

[13] Salas-Lloret D, González-Prieto R. Insights in post-translational modifications: ubiquitin and SUMO. Int J Mol Sci. 2022. 10.3390/ijms23063281. 35328702 10.3390/ijms23063281 PMC8952880

[14] Wang J, Park JS, Wei Y, Rajurkar M, Cotton JL, Fan Q, et al. TRIB2 acts downstream of wnt/tcf in liver cancer cells to regulate YAP and C/EBPα function. Mol Cell. 2013;51(2):211–25. 23769673 10.1016/j.molcel.2013.05.013 PMC4007693

[15] Du L, Li Y, Kang M, Feng M, Ren Y, Dai H, et al. USP48 is upregulated by Mettl14 to attenuate hepatocellular carcinoma via regulating SIRT6 stabilization. Cancer Res. 2021;81(14):3822–34. 33903120 10.1158/0008-5472.CAN-20-4163

[16] Zhang Y, Wang Y, Wei Y, Wu J, Zhang P, Shen S, et al. Molecular chaperone CCT3 supports proper mitotic progression and cell proliferation in hepatocellular carcinoma cells. Cancer Lett. 2016;372(1):101–9. 26739059 10.1016/j.canlet.2015.12.029

[17] Moeini A, Torrecilla S, Tovar V, Montironi C, Andreu-Oller C, Peix J, Higuera M, Pfister D, Ramadori P, Pinyol R, et al. An immune gene expression signature associated with development of human hepatocellular carcinoma identifies mice that respond to chemopreventive agents. Gastroenterology. 2019;157(5):1383–e13971311. 31344396 10.1053/j.gastro.2019.07.028 PMC6815707

[18] Long Y, Wang W, Liu S, Wang X, Tao Y. The survival prediction analysis and preliminary study of the biological function of YEATS2 in hepatocellular carcinoma. Cell Oncol (Dordrecht Netherlands). 2024;47(6):2297–316. 10.1007/s13402-024-01019-4 PMC12974064 39718737

[19] Cho YA, Choi S, Park S, Park CK, Ha SY. Expression of pregnancy up-regulated non-ubiquitous calmodulin kinase (PNCK) in hepatocellular carcinoma. Cancer Genomics Proteomics. 2020;17(6):747–55. 33099476 10.21873/cgp.20229 PMC7675661

[20] Grinchuk OV, Yenamandra SP, Iyer R, Singh M, Lee HK, Lim KH, Chow PK, Kuznetsov VA. Tumor-adjacent tissue co-expression profile analysis reveals pro-oncogenic ribosomal gene signature for prognosis of resectable hepatocellular carcinoma. Mol Oncol. 2018;12(1):89–113. 29117471 10.1002/1878-0261.12153 PMC5748488

[21] Leek JT, Johnson WE, Parker HS, Jaffe AE, Storey JD. The Sva package for removing batch effects and other unwanted variation in high-throughput experiments. Bioinformatics. 2012;28(6):882–3. 22257669 10.1093/bioinformatics/bts034 PMC3307112

[22] Van Allen EM, Miao D, Schilling B, Shukla SA, Blank C, Zimmer L, Sucker A, Hillen U, Foppen MHG, Goldinger SM, et al. Genomic correlates of response to CTLA-4 Blockade in metastatic melanoma. Sci (New York NY). 2015;350(6257):207–11. 10.1126/science.aad0095 PMC5054517 26359337

[23] Nathanson T, Ahuja A, Rubinsteyn A, Aksoy BA, Hellmann MD, Miao D, et al. Somatic mutations and neoepitope homology in melanomas treated with CTLA-4 blockade. Cancer Immunol Res. 2017;5(1):84–91. 27956380 10.1158/2326-6066.CIR-16-0019 PMC5253347

[24] Ulloa-Montoya F, Louahed J, Dizier B, Gruselle O, Spiessens B, Lehmann FF, et al. Predictive gene signature in MAGE-A3 antigen-specific cancer immunotherapy. J Clin Oncol. 2013;31(19):2388–95. 23715562 10.1200/JCO.2012.44.3762

[25] Hugo W, Zaretsky JM, Sun L, Song C, Moreno BH, Hu-Lieskovan S, et al. Genomic and transcriptomic features of response to anti-PD-1 therapy in metastatic melanoma. Cell. 2016;165(1):35–44. 26997480 10.1016/j.cell.2016.02.065 PMC4808437

[26] Riaz N, Havel JJ, Makarov V, Desrichard A, Urba WJ, Sims JS, Hodi FS, Martín-Algarra S, Mandal R, Sharfman WH, et al. Tumor and microenvironment evolution during immunotherapy with nivolumab. Cell. 2017;171(4):934–e949916. 29033130 10.1016/j.cell.2017.09.028 PMC5685550

[27] Mariathasan S, Turley SJ, Nickles D, Castiglioni A, Yuen K, Wang Y, et al. TGFβ attenuates tumour response to PD-L1 blockade by contributing to exclusion of T cells. Nature. 2018;554(7693):544–8. 29443960 10.1038/nature25501 PMC6028240

[28] Hsu CL, Ou DL, Bai LY, Chen CW, Lin L, Huang SF, Cheng AL, Jeng YM, Hsu C. Exploring markers of exhausted CD8 T cells to predict response to immune checkpoint inhibitor therapy for hepatocellular carcinoma. Liver Cancer. 2021;10(4):346–59. 34414122 10.1159/000515305 PMC8339511

[29] Wilkerson MD, Hayes DN. ConsensusClusterPlus: a class discovery tool with confidence assessments and item tracking. Bioinformatics. 2010;26(12):1572–3. 20427518 10.1093/bioinformatics/btq170 PMC2881355

[30] Wu T, Hu E, Xu S, Chen M, Guo P, Dai Z, et al. Clusterprofiler 4.0: a universal enrichment tool for interpreting omics data. Innovation (Camb). 2021;2(3):100141. 34557778 10.1016/j.xinn.2021.100141 PMC8454663

[31] Langfelder P, Horvath S. WGCNA: an R package for weighted correlation network analysis. BMC Bioinformatics. 2008;9:559. 19114008 10.1186/1471-2105-9-559 PMC2631488

[32] Zeng D, Ye Z, Shen R, Yu G, Wu J, Xiong Y, Zhou R, Qiu W, Huang N, Sun L, et al. IOBR: Multi-Omics Immuno-Oncology biological research to Decode tumor microenvironment and signatures. Front Immunol. 2021;12:687975. 34276676 10.3389/fimmu.2021.687975 PMC8283787

[33] Li K, Zhang R, Wen F, Zhao Y, Meng F, Li Q, et al. Single-cell dissection of the multicellular ecosystem and molecular features underlying microvascular invasion in HCC. Hepatology. 2024;79(6):1293–309. 37972953 10.1097/HEP.0000000000000673 PMC11095903

[34] Wu R, Guo W, Qiu X, Wang S, Sui C, Lian Q, et al. Comprehensive analysis of spatial architecture in primary liver cancer. Sci Adv. 2021;7(51):eabg3750. 34919432 10.1126/sciadv.abg3750 PMC8683021

[35] Wang LT, Wang SN, Chiou SS, Liu KY, Chai CY, Chiang CM, Huang SK, Yokoyama KK, Hsu SH. TIP60-dependent acetylation of the SPZ1-TWIST complex promotes epithelial-mesenchymal transition and metastasis in liver cancer. Oncogene. 2019;38(4):518–32. 30154425 10.1038/s41388-018-0457-z PMC6345675

[36] Wei Z, Jia J, Heng G, Xu H, Shan J, Wang G, et al. Sirtuin-1/mitochondrial ribosomal protein S5 axis enhances the metabolic flexibility of liver cancer stem cells. Hepatology. 2019;70(4):1197–213. 30901096 10.1002/hep.30622

[37] Lao Y, Cui X, Xu Z, Yan H, Zhang Z, Zhang Z, et al. Glutaryl-CoA dehydrogenase suppresses tumor progression and shapes an anti-tumor microenvironment in hepatocellular carcinoma. J Hepatol. 2024;81(5):847–61. 38825017 10.1016/j.jhep.2024.05.034

[38] Lim JTC, Kwang LG, Ho NCW, Toh CCM, Too NSH, Hooi L, Benoukraf T, Chow PK, Dan YY, Chow EK, et al. Hepatocellular carcinoma organoid co-cultures mimic angiocrine crosstalk to generate inflammatory tumor microenvironment. Biomaterials. 2022;284:121527. 35483200 10.1016/j.biomaterials.2022.121527

[39] Sun J, Ding J, Shen Q, Wang X, Wang M, Huang Y, et al. Decreased propionyl-CoA metabolism facilitates metabolic reprogramming and promotes hepatocellular carcinoma. J Hepatol. 2023;78(3):627–42. 36462680 10.1016/j.jhep.2022.11.017

[40] Zhang Y, Xie M, Wen J, Liang C, Song Q, Liu W, Liu Y, Song Y, Lau HCH, Cheung AH, et al. Hepatic TM6SF2 activates antitumour immunity to suppress metabolic dysfunction-associated steatotic liver disease-related hepatocellular carcinoma and boosts immunotherapy. Gut. 2025;74(4):639–51. 39667906 10.1136/gutjnl-2024-333154 PMC12014897

[41] Wen J, Zhang X, Wong CC, Zhang Y, Pan Y, Zhou Y, et al. Targeting squalene epoxidase restores anti-PD-1 efficacy in metabolic dysfunction-associated steatohepatitis-induced hepatocellular carcinoma. Gut. 2024;73(12):2023–36. 38744443 10.1136/gutjnl-2023-331117 PMC11671884

[42] Barsch M, Salié H, Schlaak AE, Zhang Z, Hess M, Mayer LS, et al. T-cell exhaustion and residency dynamics inform clinical outcomes in hepatocellular carcinoma. J Hepatol. 2022;77(2):397–409. 35367533 10.1016/j.jhep.2022.02.032

[43] Yarchoan M, Gane EJ, Marron TU, Perales-Linares R, Yan J, Cooch N, Shu DH, Fertig EJ, Kagohara LT, Bartha G, et al. Personalized neoantigen vaccine and pembrolizumab in advanced hepatocellular carcinoma: a phase 1/2 trial. Nat Med. 2024;30(4):1044–53. 38584166 10.1038/s41591-024-02894-y PMC11031401

[44] Llovet JM, Kudo M, Merle P, Meyer T, Qin S, Ikeda M, et al. Lenvatinib plus pembrolizumab versus lenvatinib plus placebo for advanced hepatocellular carcinoma (LEAP-002): a randomised, double-blind, phase 3 trial. Lancet Oncol. 2023;24(12):1399–410. 38039993 10.1016/S1470-2045(23)00469-2

