# Supplementary material for: Integrating bulk RNA-seq, scRNA-seq, and spatial transcriptomics data to identify novel post-translational modification-related molecular subtypes and therapeutic responses in hepatocellular carcinoma
Source: Cancer Cell Int. 2025 Oct 3;25:330. doi: 10.1186/s12935-025-03964-y (PMC12495838; doi:10.1186/s12935-025-03964-y)
Supplement: Supplementary file 1 — Supplementary Material 1. [file 12935_2025_3964_MOESM1_ESM.docx]

**Materials and methods**

**Machine learning**

The integration of 10 machine-learning algorithms was conducted. Using 10-fold cross-validation approaches, LASSO, ridge, stepwise Cox, CoxBoost, random forest (RSF), elastic network (Enet), partial least squares regression for Cox (plsRcox), supervised principal components (SuperPC), support vector machine (-SVM), and generalized boosted regression modeling (GBM) were randomly combined, and 101 combinational algorithms were generated. A high-throughput screening was performed to determine the best signature. Using the R package “randomForestSRC”, an RSF algorithm was performed. Two parameters were pivotal for RFS, including ntree and mtry. The ntree refers to the number of trees in the forest, while the mtry refers to the number of variables for splitting at each node. A grid search on ntree and mtry was performed using 10-fold cross-validation. All the pairs of (ntree, mtry) were generated, and the one with the best C-index value was then screened out as the optimized parameter. Using the R package “glmnet”, the ridge algorithms, Enet, and LASSO were performed. Lambda was identified by 10-fold cross-validation. The L1-L2 trade-off parameter, ꬰ, was set to 0-1 (interval = 0.1). Using the R package“”, the stepwise Cox model was used. A stepwise algorithm based on the AIC (Akaike information criterion) was employed. To fit a Cox proportional hazards model, component-wise likelihood-based boosting was performed based on the R package “CoxBoost”. The 10-fold cross-validation routine “optimCoxBoostPenalty” function was utilized for first determining the optimal penalty (amount of shrinkage). After the parameter was determined, the other tuning parameter of the algorithm was screened out using the function “cv.CoxBoost”. The principal routine CoxBoost was applied to select the dimension of the multivariate Cox model. Using the “plsRcox” package, the plsRcox model was applied. The “cv.plsRcox” function was employed for the determination of the number of components requested. The “plsRcox” function was utilized to fit a partial least squares regression generalized linear model. Using the “SUPERPC” package, the SuperPC model was applied. The SuperPC model refers to a principal component analysis that generates a liner combination of the features or variables of interest that capture the directions of the largest variation in a dataset. To estimate the optimal feature threshold in SUPERPC, the “superpc.cv” function based on 10-fold cross-validation was employed. By employing the “pre-validation” function, problems with fitting Cox models to small validation datasets can be avoided. Using the R package “gbm”, the GBM algorithm was applied. The 10-fold cross-validation enables the “cv.gbm” function to select an index for number trees with minimum cross-validation error. The fit of the generalized boosted regression model was performed based on the gbm function. Using the package “svm”, the SVM model was applied. The regression approach incorporates the censoring concept when determining the constraints on inequality in the support vector problem.

**Single-cell RNA sequencing (scRNA-seq)**

The Seurat package played a crucial role in the processing of scRNA-seq data. It encompassed quality control, statistical analysis, and data exploration. For quality control, genes detected in more than 3 cells were retained, while cells meeting the criteria of having over 200 detected genes and mitochondrial expression genes comprising no more than 5% were included. Principal Component Analysis (PCA) was employed to distinguish relevant dimensions, with a significance threshold of p < 0.05. Following this, the T-distributed random neighbor Embedding (tSNE) algorithm was utilized to achieve dimensionality reduction and cluster analysis. Differential expression analysis was executed through the limma package to identify marker genes for each cluster. Selection criteria involved a p value of < 0.05 and an absolute log2 fold change greater than 0.5. Subsequently, the singleR package, informed by marker gene populations, was used to label different cell clusters. These labels were then subjected to manual verification and correction using the CellMarker database.

**Spatial transcriptomics (ST)**

ST HCC data was downloaded from the PMID: 34919432. In total, 3 sections from separate individuals, recovering a total number of 8402 spots containing tissue sequenced to 43,648 post-normalizations mean reads per spot, were chosen based on H&E image data. Then, the selected data was converted into a Seurat object using InputFromTable (STutility) for normalization, quality control, dimensionality reduction, and Louvain clustering in addition to custom scripts. Spots expressing less than 200 features were excluded from downstream analysis. Normalization across spots was performed using the SCTransform function. Dimensionality reduction and clustering was performed with principal components analysis (PCA) and Uniform manifold approximation and projection (UMAP). Differentially expressed genes were performed using the FindAllMarkers function in Seurat with average log2FC > 0.3 and adjusted P-value < 0.05 (two-sided Wilcoxon rank-sum test with a Bonferroni correction). Spatial feature expression plots were generated with the STUtility R package (version 1.0.0), ggplot2 (Version 3.4.0) and ggrepl R packages (Version 0.9.2). Pearson correlation was calculated and hierarchical clustering of ST clusters was performed in the pheatmap R package. Signature scoring derived from SC (see below) or ST RMI signatures was performed with the AddModuleScore function in Seurat with default parameters.
