# Supplementary figures and images for: Integrating bulk RNA-seq, scRNA-seq, and spatial transcriptomics data to identify novel post-translational modification-related molecular subtypes and therapeutic responses in hepatocellular carcinoma
Source: Cancer Cell Int. 2025 Oct 3;25:330. doi: 10.1186/s12935-025-03964-y (PMC12495838; doi:10.1186/s12935-025-03964-y)

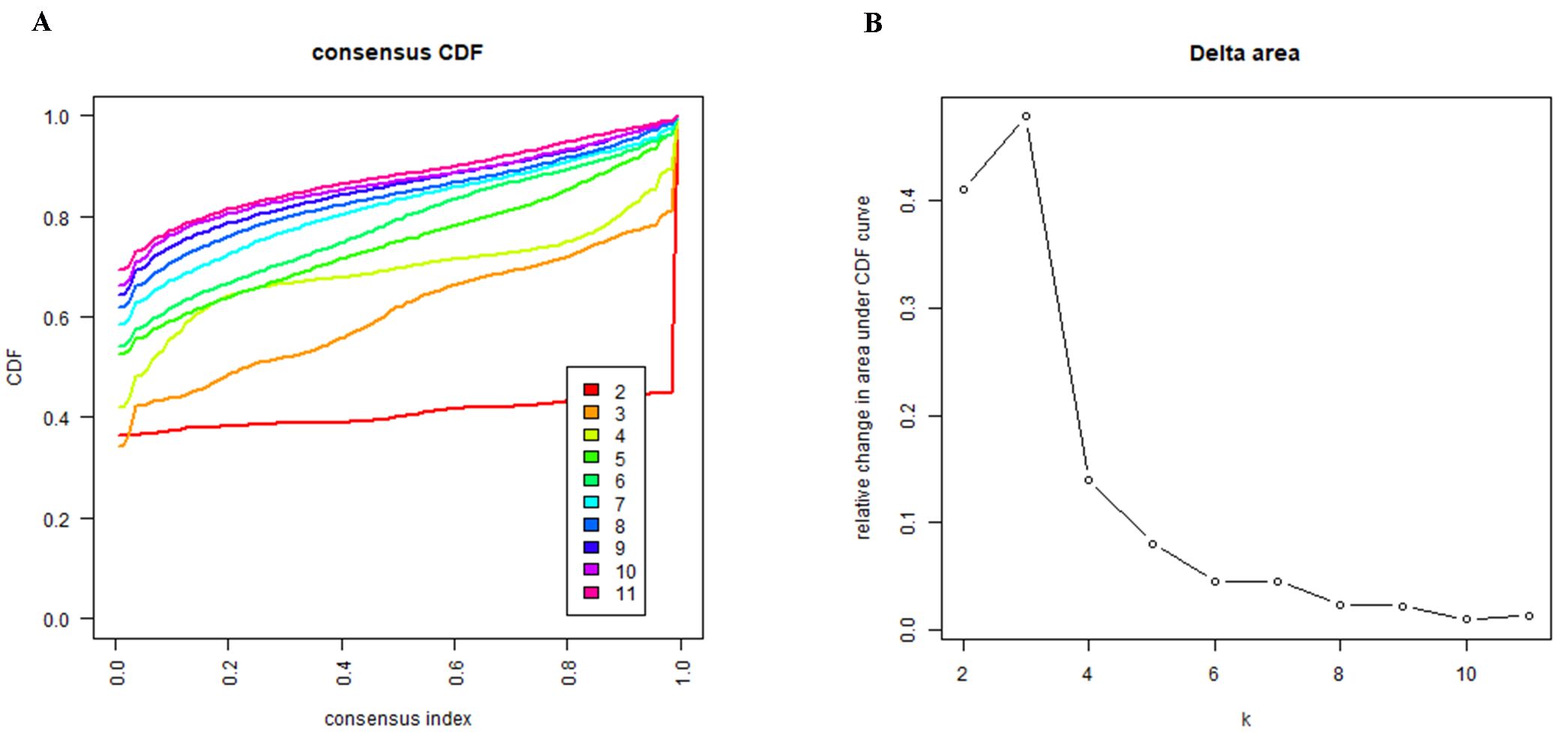

Supplement: Supplementary file 6 — Supplementary Material 6: (A)The CDF curves of consensus matrix for each k. (B) The delta area of consensus matrix for each k. [file 12935_2025_3964_MOESM6_ESM.tif]

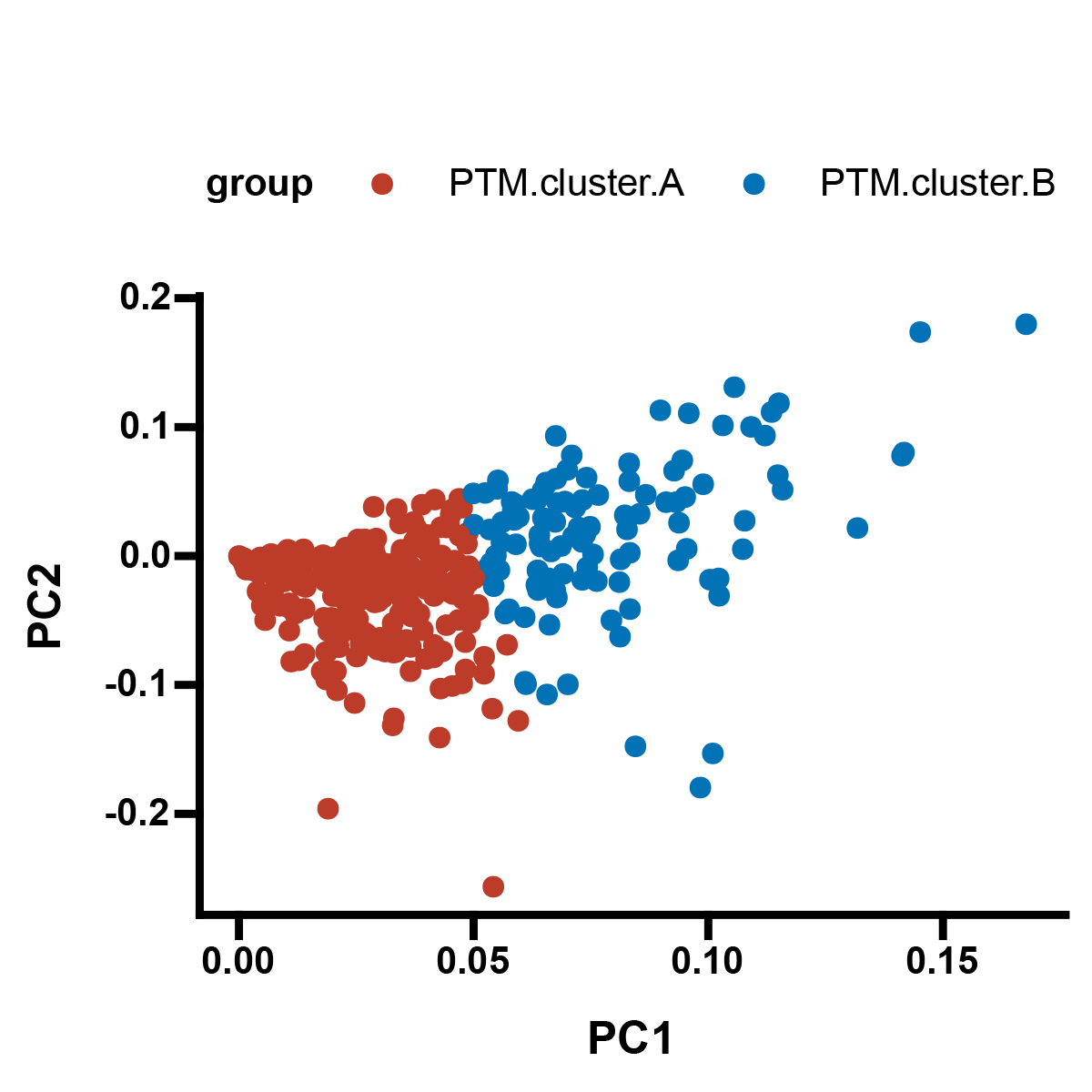

Supplement: Supplementary file 7 — Supplementary Material 7: PCA analysis [file 12935_2025_3964_MOESM7_ESM.tif]

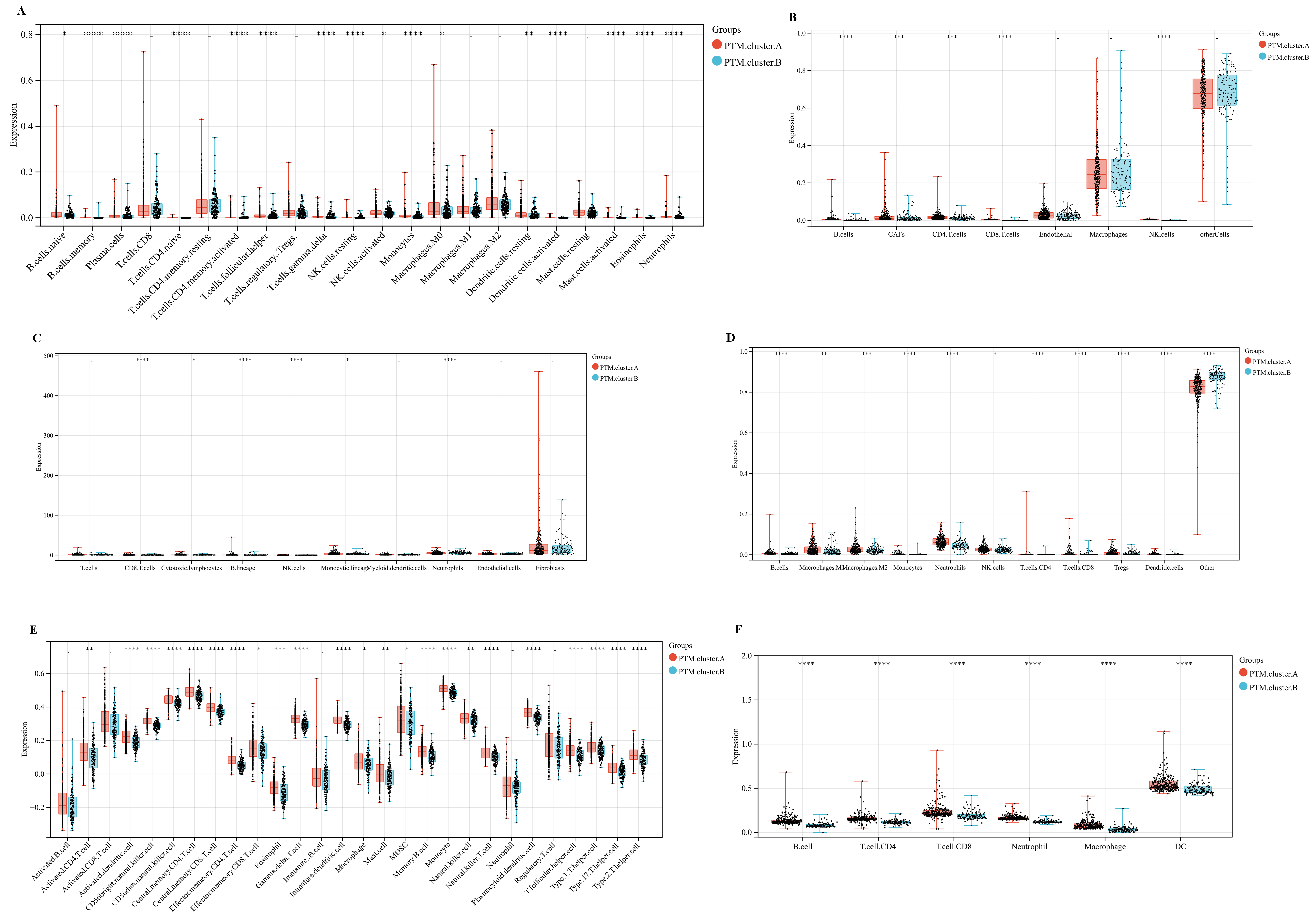

Supplement: Supplementary file 8 — Supplementary Material 8: The different immune infiltration between the molecular subtypes by using CIBERSORT-ABS (A), EPIC (B), MCPcounter (C), quanTIseq (D), ssGSEA (E), TIMER (F) (*p<0.05，**p<0.01，***p< 0.001). [file 12935_2025_3964_MOESM8_ESM.tif]

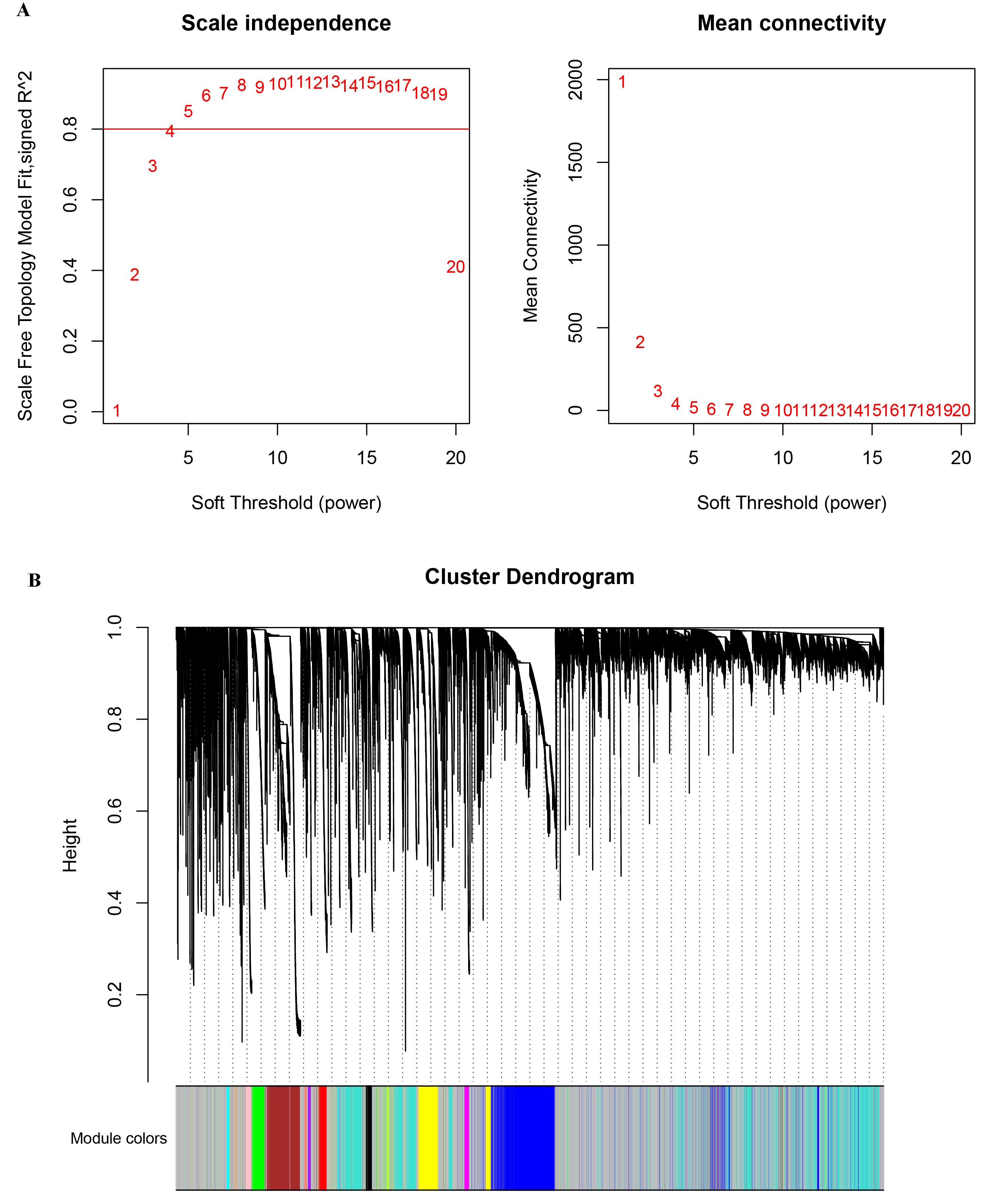

Supplement: Supplementary file 9 — Supplementary Material 9: (A) The sample similarity of each subgroup was assessed by calculating the Silhoutte score. (B) Identification of co-expression gene modules [file 12935_2025_3964_MOESM9_ESM.tif]

iAUC\_sh

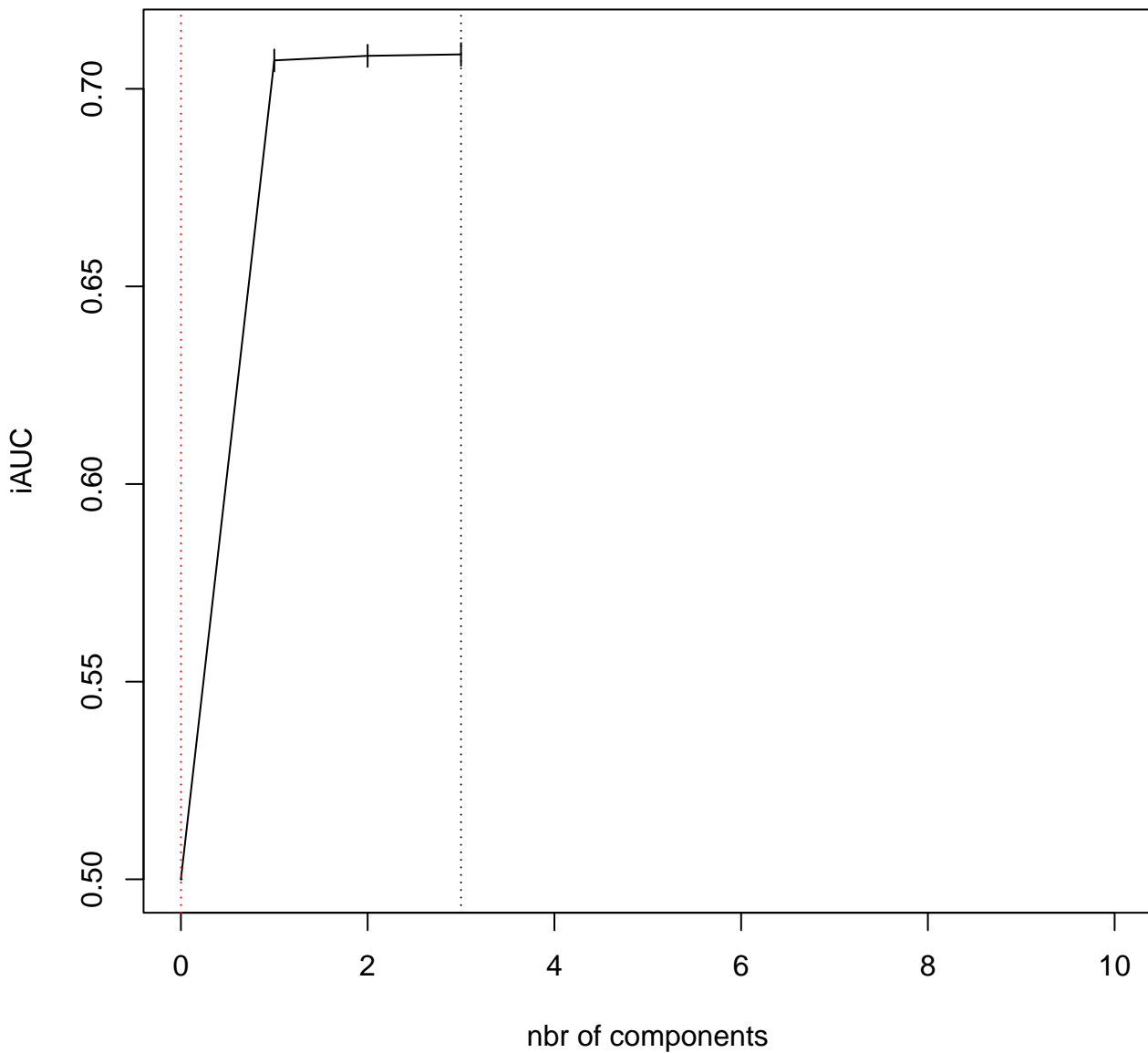

iAUC\_sh

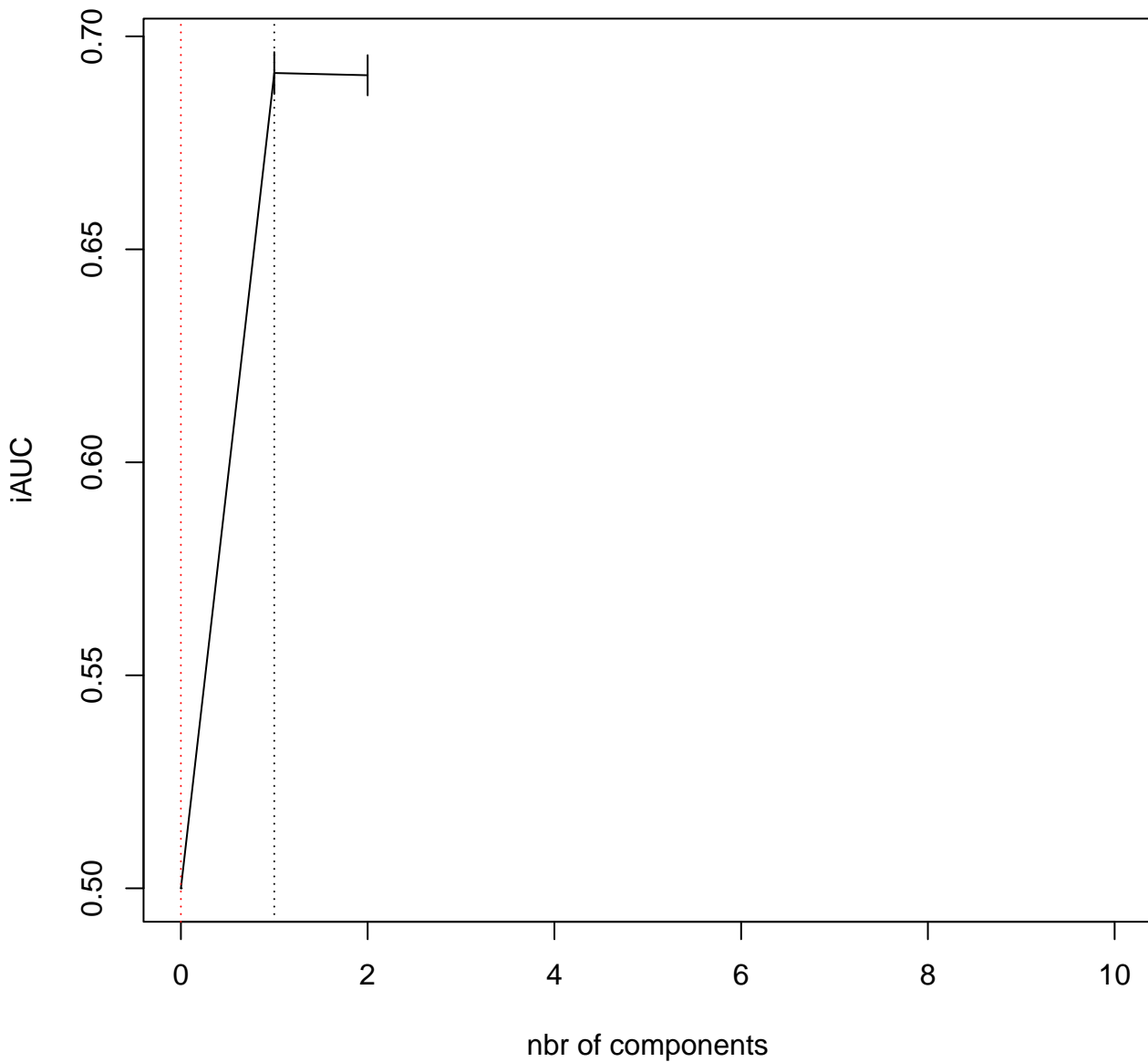

iAUC\_sh

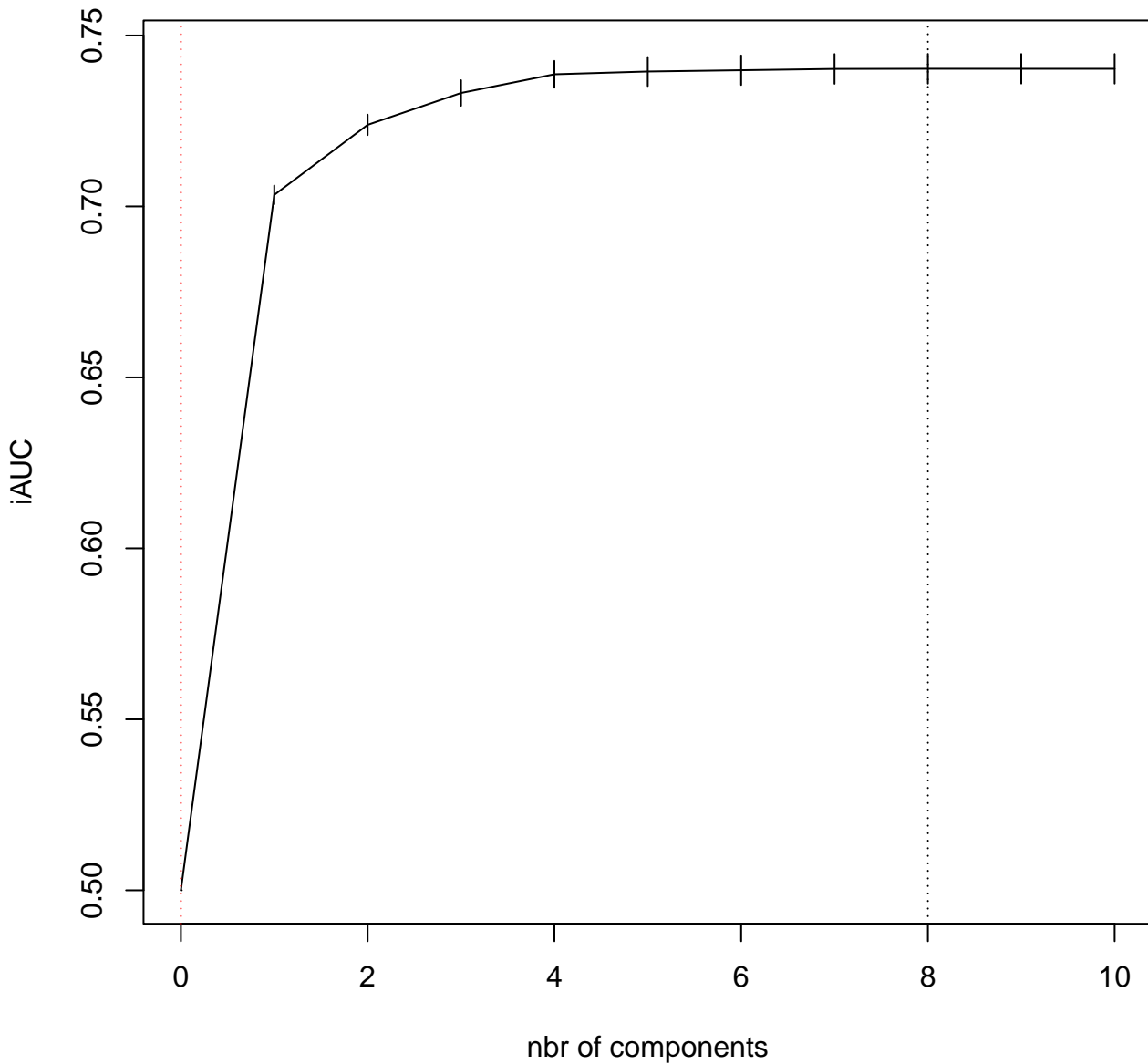

iAUC\_sh

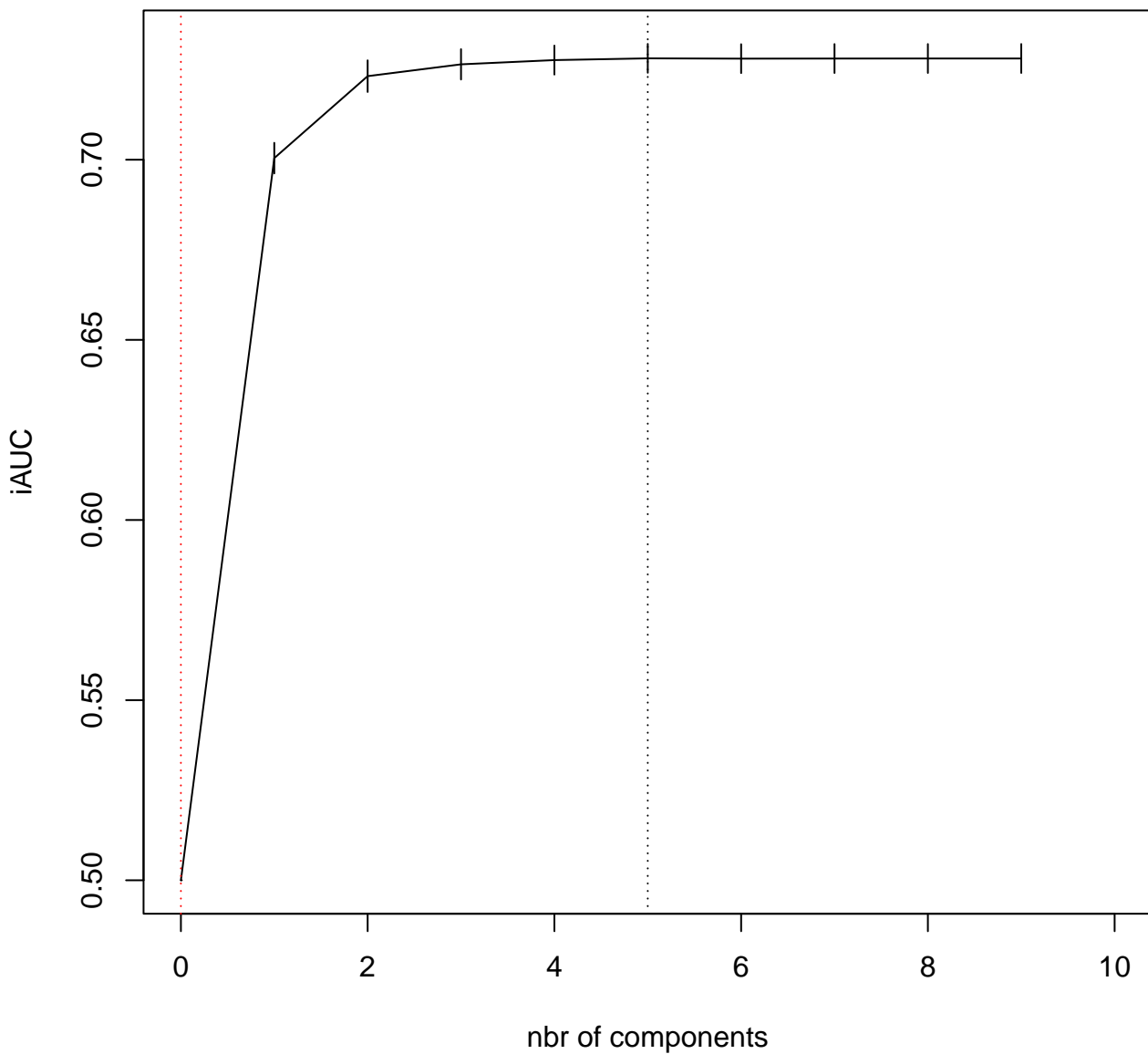

iAUC\_sh

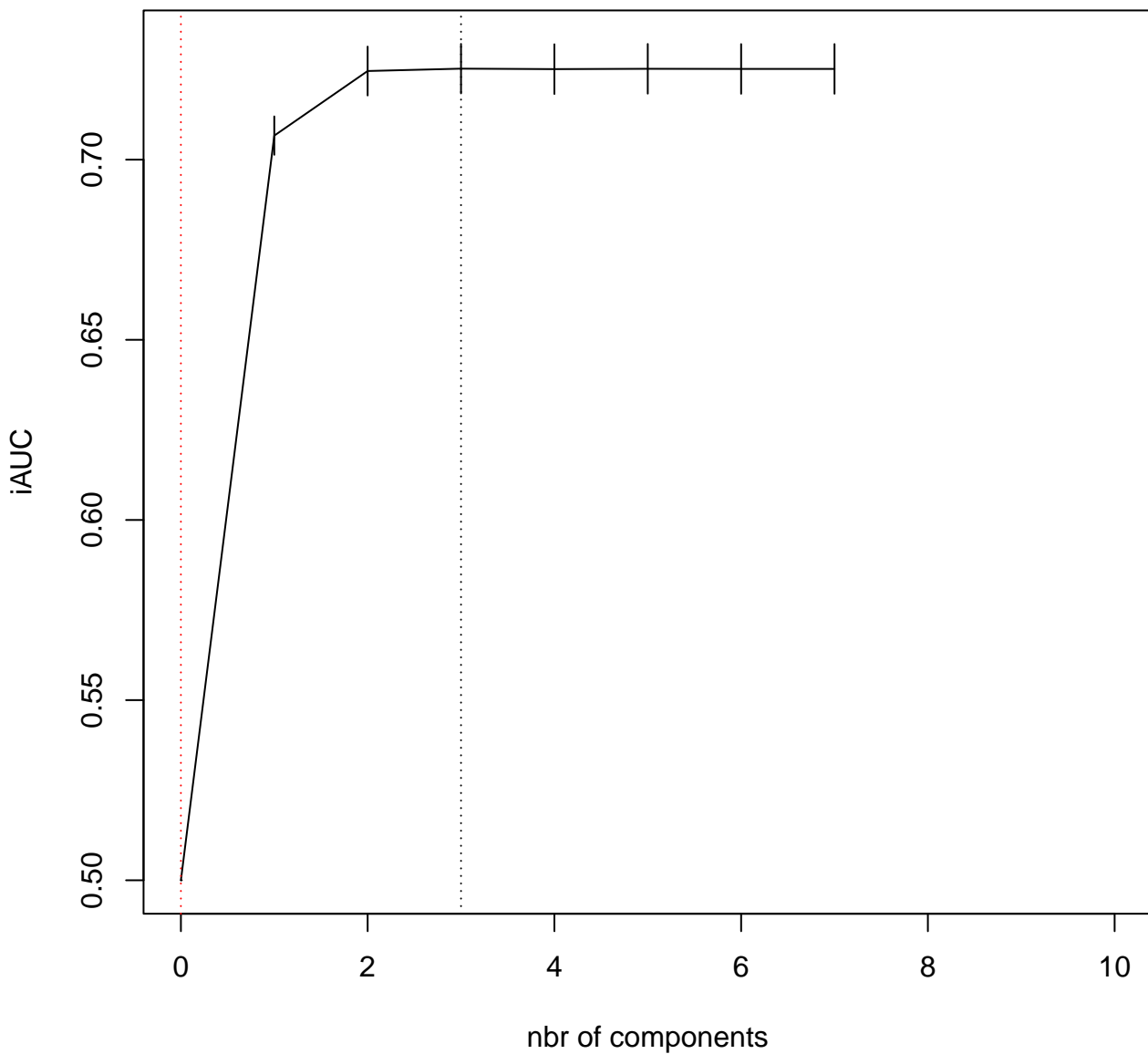

iAUC\_sh

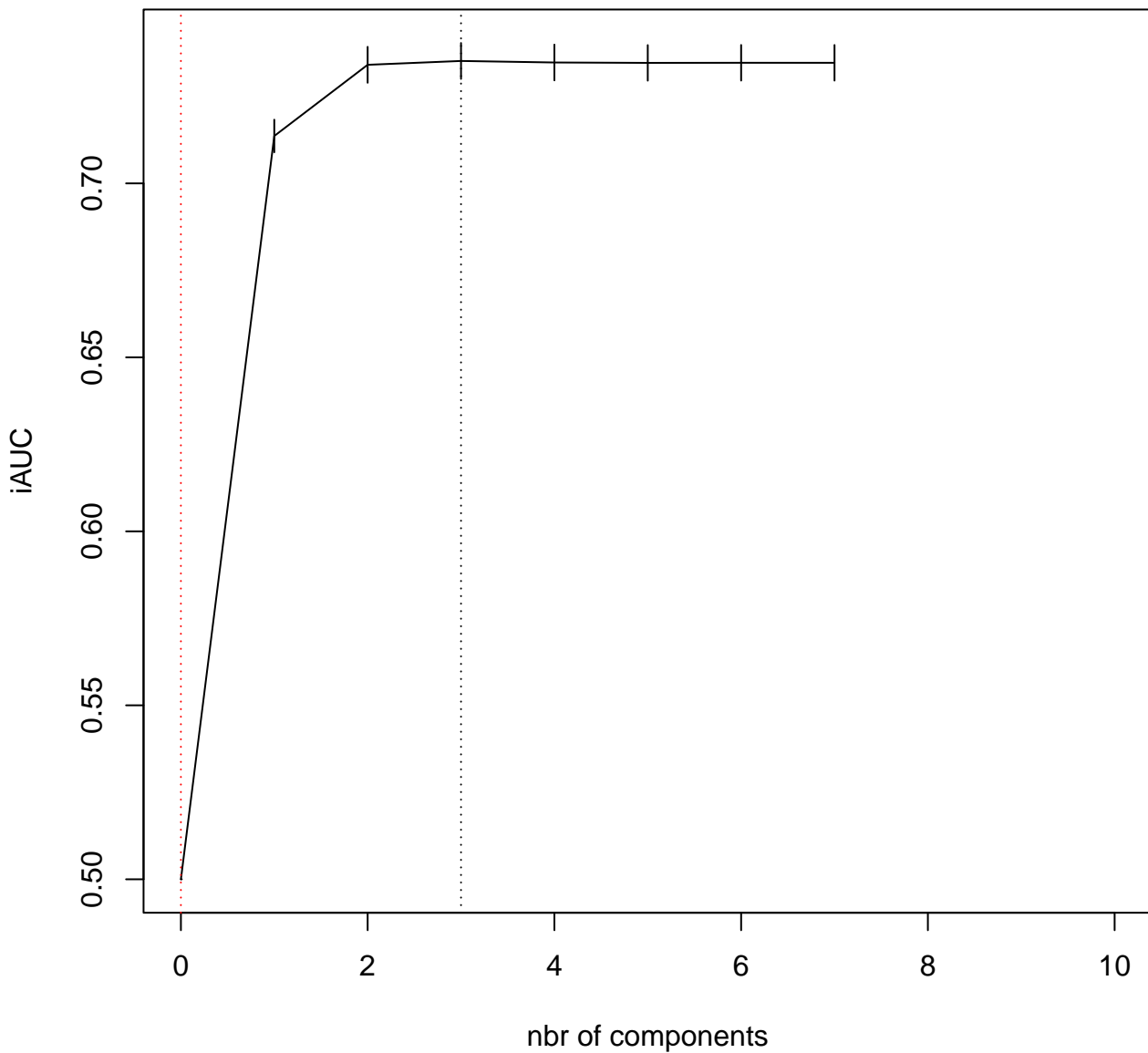

Supplement: Supplementary file 10 — Supplementary Material 10: The cross-validation details of machine learning [file 12935_2025_3964_MOESM10_ESM.pdf]

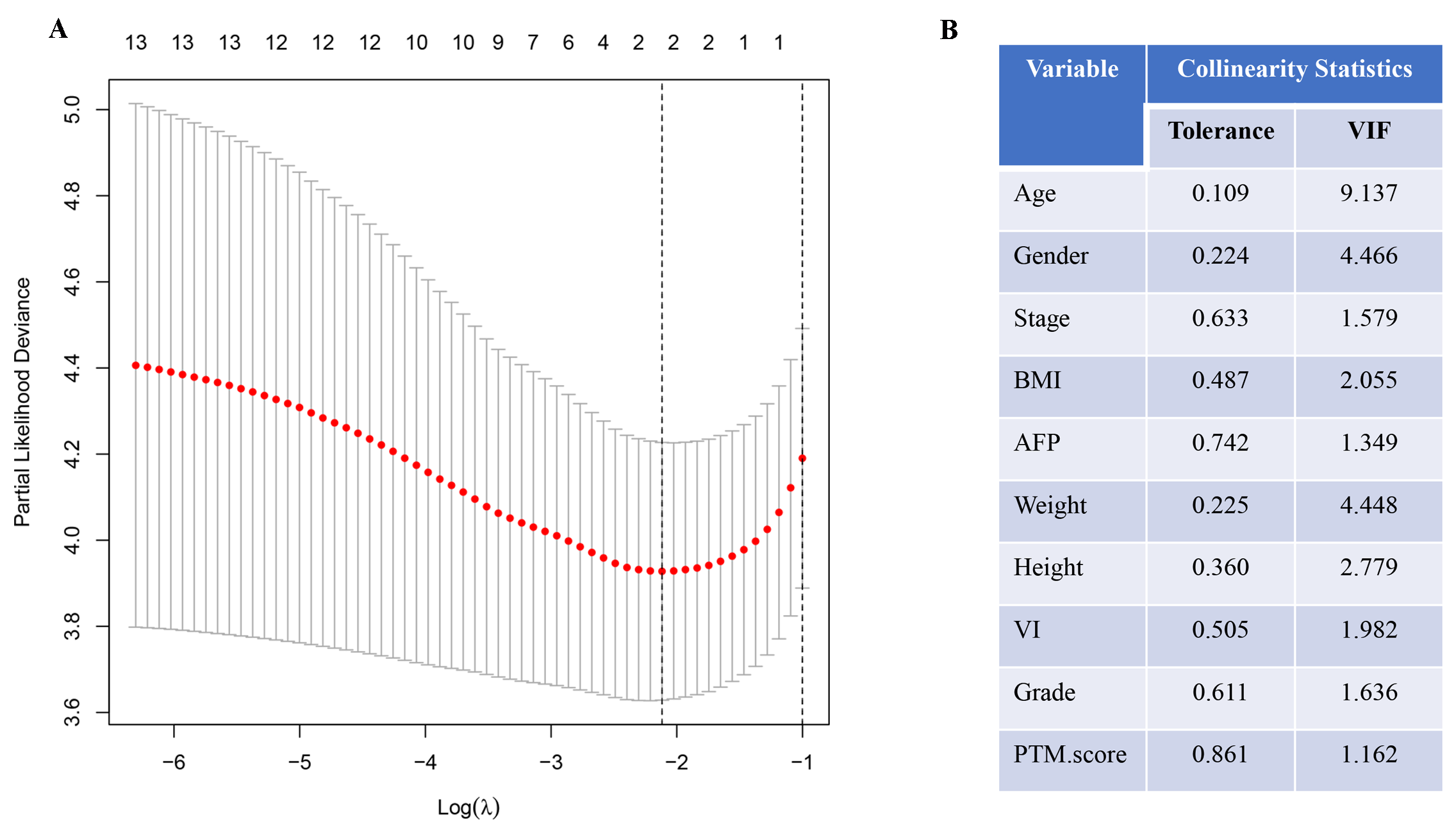

Supplement: Supplementary file 11 — Supplementary Material 11: (A) LASSO analysis. (B) Collinearity analysis of risk factors [file 12935_2025_3964_MOESM11_ESM.tif]

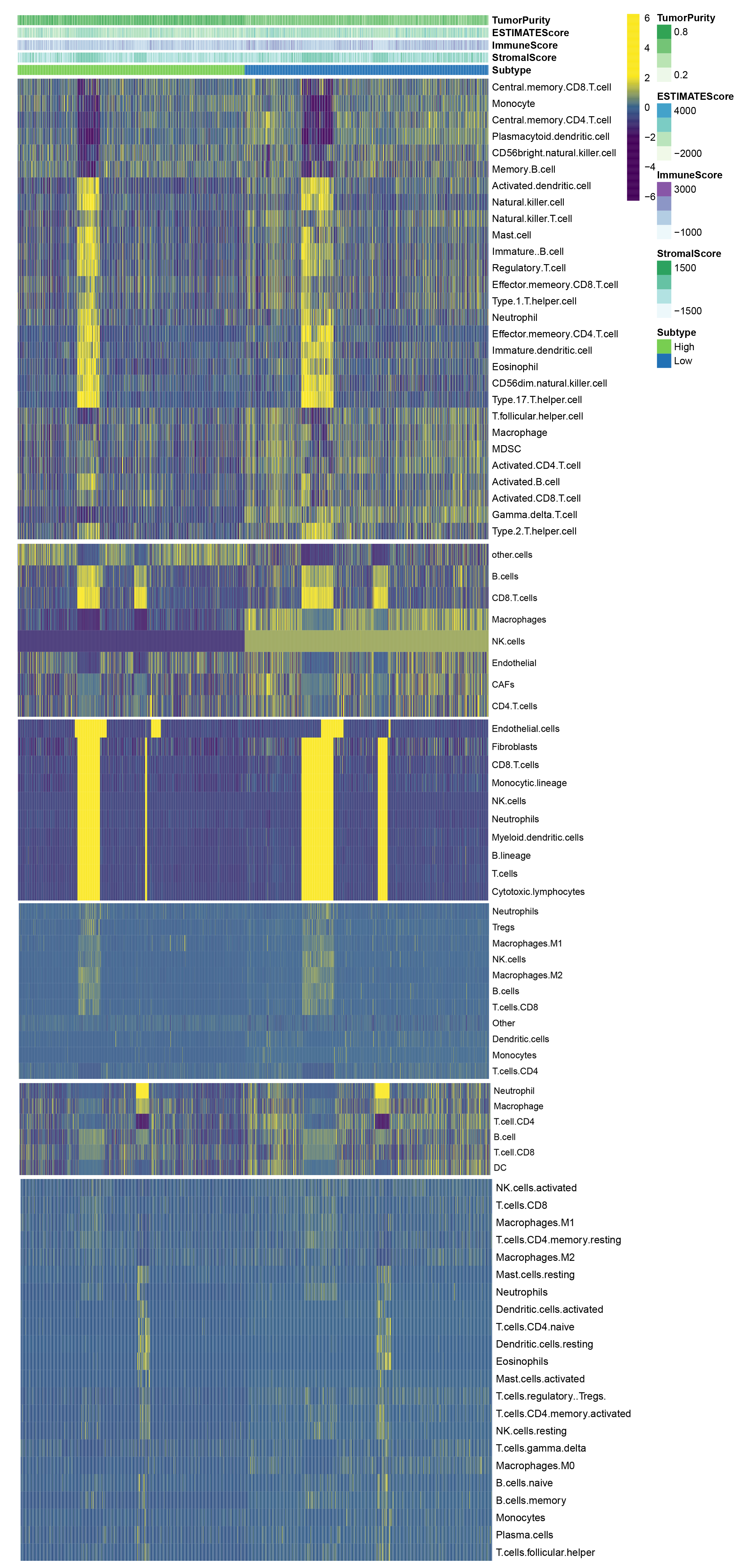

Supplement: Supplementary file 12 — Supplementary Material 12: Immune cell infiltration of high PTM.score and low PTM.score groups [file 12935_2025_3964_MOESM12_ESM.tif]

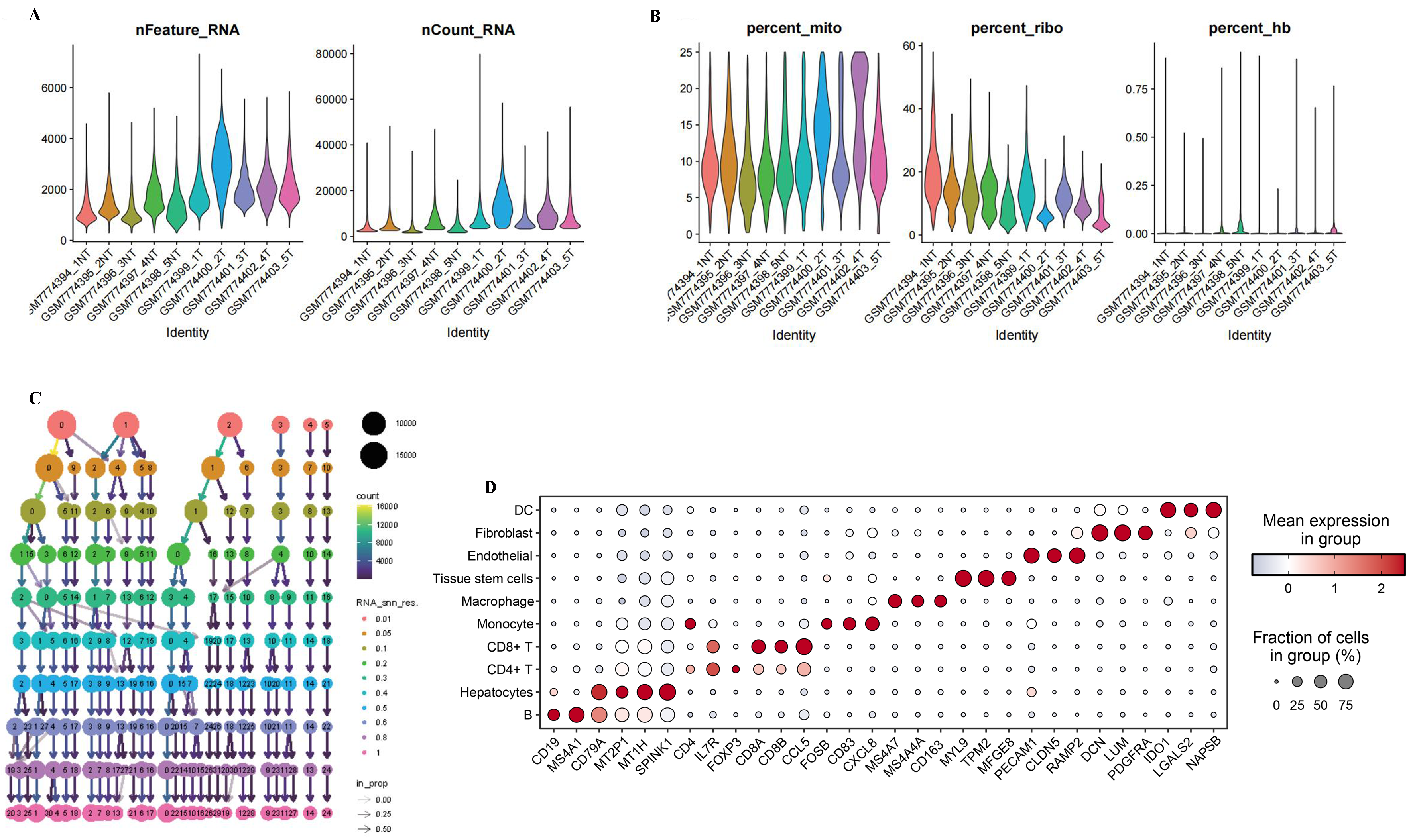

Supplement: Supplementary file 13 — Supplementary Material 13: Analysis of single-cell RNA sequencing data. (A, B) Violin diagram showed the threshold to filter cells. (C) Clustree showed the distinct clusters. (D) Dotplot of marker genes for cell types [file 12935_2025_3964_MOESM13_ESM.tif]
